# Comparative Genomics Revealed Wide Intra-Species Genetic Heterogeneity and Lineage-Specific Genes of *Akkermansia muciniphila*

**DOI:** 10.1128/spectrum.02439-21

**Published:** 2022-05-10

**Authors:** Weicheng Li, Jiaqi Sun, Yongjian Jing, Jie Zhao, Qiong Wu, Jiaqi Liu, Lai-Yu Kwok, Wenguang Zhang, Zhihong Sun, Zhi Zhong, Wenjun Liu

**Affiliations:** a Key Laboratory of Dairy Biotechnology and Engineering (IMAU), Ministry of Education; Key Laboratory of Dairy Products Processing, Ministry of Agriculture and Rural Affairs; Inner Mongolia Key Laboratory of Dairy Biotechnology and Engineering; Collaborative Innovative Center of Ministry of Education for Lactic Acid Bacteria and Fermented Dairy Products, Inner Mongolia Agricultural University, Hohhot, China; b Center of Information and Network Technology, Inner Mongolia Agricultural University, Hohhot, China; c Inner Mongolia Engineering Research Center of Genomic Big Data for Agriculture, Inner Mongolia Agricultural University, Hohhot, China; National Health Research Institutes

**Keywords:** *Akkermansia muciniphila*, carbohydrate metabolism, gut microbe, next-generation probiotics, recombination

## Abstract

Akkermansia muciniphila has potential as a next-generation probiotic, but few previous studies attempted to analyze its intraspecies population diversity. In this study, we performed a comparative genomic analysis of 112 filtered genomes from the NCBI database. The populations formed three clades (A-C) on the phylogenetic tree, suggesting the existence of three genetic lineages though clades B and C were phylogenetically closer than clade A. The three clades also showed geographic-based clustering, different genetic characteristics, and clade-specific genes. Two putative functional genes (*RecD2* and *xerD*) were specific to clade C due to genomic islands. These lineage-specific genes might be associated with differences in genomic features (number of phages/genomic islands, pan-core genome, recombination rate, genetic diversity) between genetic lineages. The carbohydrate utilization gene profile (particularly for glycolytic hydrolases and carbohydrate esterases) also varied between clades, suggesting different carbohydrate metabolism potential/requirements between genetic lineages. Our findings provide important implications for future research on *A. muciniphila*.

**IMPORTANCE** Akkermansia muciniphila has been widely accepted as part of the next generation of probiotics. However, most current studies on *A. muciniphila* have focused on the application of type strain BAA835^T^ in the treatment of diseases, while few studies have reported on the genomic specificity, population structure, and functional characteristics of *A. muciniphila* species. By comparing the genomes of 112 strains from NCBI which met the quality control conditions, we found that the *A. muciniphila* population could be divided into three main clades (clades A to C) and presented a certain regional aggregation. There are significant differences among the three clades in their genetic characteristics and functional genes (the type strain BAA835^T^ was located in clade A), especially in genes related to carbohydrate metabolism. It should be mentioned that probiotics should be a concept at the strain level rather than at the gut species level, so the probiotic properties of *A. muciniphila* need to be carefully interpreted.

## INTRODUCTION

As a potential next-generation probiotic species, Akkermansia muciniphila has attracted much attention due to its unique characteristics (1, 2). As a common species in the human gut, *A. muciniphila* plays an important role in maintaining a healthy and intact intestinal mucus layer (3), thereby reducing translocation of proinflammatory lipopolysaccharides, regulating adipose tissue metabolism, decreasing insulin resistance, and maintaining glucose homeostasis (4–6). In addition, *A. muciniphila* maintains a stable intestinal microbial community, enhancing the host’s immunity, which in turn helps fight cancer (7) and alleviates other medical conditions such as epilepsy (8) and amyotrophic lateral sclerosis (9). Despite the promising probiotic features of *A. muciniphila*, some of its negative aspects are worth noting. For example, significant positive associations have been reported between *A. muciniphila* and certain metabolic diseases (10) and type 2 diabetes (11). The contrasting characteristics of *A. muciniphila* have thus caused much debate about the prospect of applying it as a probiotic. In view of the clinical significance of the gut microbiota in maintaining colonic homeostasis and host health, it is important to explore novel probiotics; therefore, extensive research is necessary to expand our knowledge of candidate next-generation probiotics such as *A. muciniphila*.

Unlike some other popular and traditional probiotic species, *A. muciniphila* has only been explored in depth by a few studies, particularly from a functional genomics perspective. The *Akkermansia* genus is a new member of the Verrucomicrobia phylum (12), and *A. muciniphila* was first isolated from human feces as a new mucus-degrading bacterium in 2004 (3). High-throughput genomic analyses have allowed us to systematically and effectively compare the entire genomes of hundreds of individual strains of a species of interest. Such an approach has been proven to be extremely valuable in revealing key insights into the population structures of species of interest and discovering health-promoting elements encoded in bacterial genomes. The genome sequence of ATCC BAA-835^T^ became the first available *A. muciniphila* strain in 2011 (13). Based on genomic and phylogenetic analyses, Guo et al. (14) divided the then-available *A. muciniphila* strains into three phylogenetic groups which exhibited different metabolic and functional characteristics, although one obvious drawback of the study was that the average nucleotide identity (ANI) employed in the study was lower than the standard species-level cutoff of 95% (14, 15). Xing et al. (16) comparatively analyzed 23 *Akkermansia* genomes from different strains and identified 4 clades in the phylogenetic tree. The *A. muciniphila* strains isolated from diverse geographical regions and ecological niches formed closely related clades. Karcher et al. (17) uncovered a large phylogenetic and functional diversity of human-originating *Akkermansia*. However, this study was limited to genus-level analysis and did not focus on the species of interest, *A. muciniphila*. The large interspecies differences cover the intraspecies differences such that that the population structures, genetic backgrounds, and functional characteristics within the species *A. muciniphila* cannot be well described.

ANI has been widely accepted as a classical species classification method (15), and two strains with a shared ANI greater than 95% are considered to be the same species. In this study, 112 whole-genome sequences of *A. muciniphila* sharing more than 95% ANI with the model strain ATCC BAA-835^T^ were selected from the NCBI Refseq database for comparative genomic studies. Phylogenetic analysis showed that the *A. muciniphila* species consisted of two lineages composed of three clades, and the three clades all showed obvious separate aggregation. The three genetic clades showed significant differences in genetic background, genomic characteristics (genome size and GC content), and functional genomic characteristics (especially the number of glycoside hydrolase family genes). The *RecD2* (ATP-dependent *RecD*-like DNA helicase) and *xerD* (tyrosine recombinase) genes, which are unique to clade C, led to a high proportion of core genes in the branch and a recombination rate of approximately zero, a low genetic diversity, and a greater number of phages. Insights into the evolutionary history, population structures, gene clusters, and carbohydrate-related genes of *A. muciniphila* provide more information about the possible physiological and probiotic mechanisms of *A. muciniphila* strains. This manuscript provides some instructions for in-depth research into the use of *A. muciniphila* as a gut probiotic in the future.

## RESULTS

### Genomic characterization of *Akkermansia muciniphila* at the species level.

As of September 2020, NCBI’s Refseq database contains genomic data for 130 strains (Table S1 in the supplemental material). To confirm the genomic taxonomic status of *A. muciniphila*, we performed paired ANI analysis on the 130 strains, which consisted of four species groupings (Fig. S1A). The results of phylogenetic analysis based on the core genes were consistent with the results of paired ANI analysis (Fig. S1B): 18 isolates showed interspecies differences with the other 112 isolates (including the type strain ATCC BAA-835^T^). Due to the large differences between different species, 18 strains which might have incorrect taxonomic status were removed from this study and 112 strains which shared more than 95% ANI with the type strain ATCC BAA-835^T^ were further analyzed.

Of the 112 fecal isolates, 44 human isolates were from America (39.28%), 29 were from China (25.89%), 20 were from South Korea (17.86%), and 8 isolates were from other non-human mammals (7.14%). At the species level, the *A. muciniphila* genome is 2.65 ± 0.08 Mb in size and its GC content is 55.29% ± 0.37%. There were 2,234 ± 93 coding sequences (CDS) in *A. muciniphila*. Further analysis showed a significant linear relationship between genome size and the number of CDS (R^2^ = 0.92, Fig. S2A), but no significant linear relationship between genome size and GC content (R^2^ = 0.48, Fig. S2B) or between GC content and CDS (R^2^ = 0.49, Fig. S2C). According to the fitted linear function, the average genome length of CDS was about 896.87 bp.

### Pan- core genome and phylogenetic relationships of *Akkermansia muciniphila*.

In order to facilitate in-depth evolutionary and population genetics analyses of these genomes, we initially annotated these 112 genomes. By constructing the pan-core gene set of *A. muciniphila* species, we obtained a pan-core gene set of 9,419 genes (Fig. S2), including the 1,132 core genes present in all strains, 401 soft-core genes (95% ≤ strains < 100%), 1,201 shell genes (15% ≤ strains < 95%) and 6,685 cloud genes (0% ≤ strains < 15%). The results showed that the pan-genome was open, indicating that the *A. muciniphila* strain had strong adaptability. In addition, cloud genes occupy a large proportion of the pan-genome of *A. muciniphila* and are the most important component of the pan-genome.

**FIG 1:**
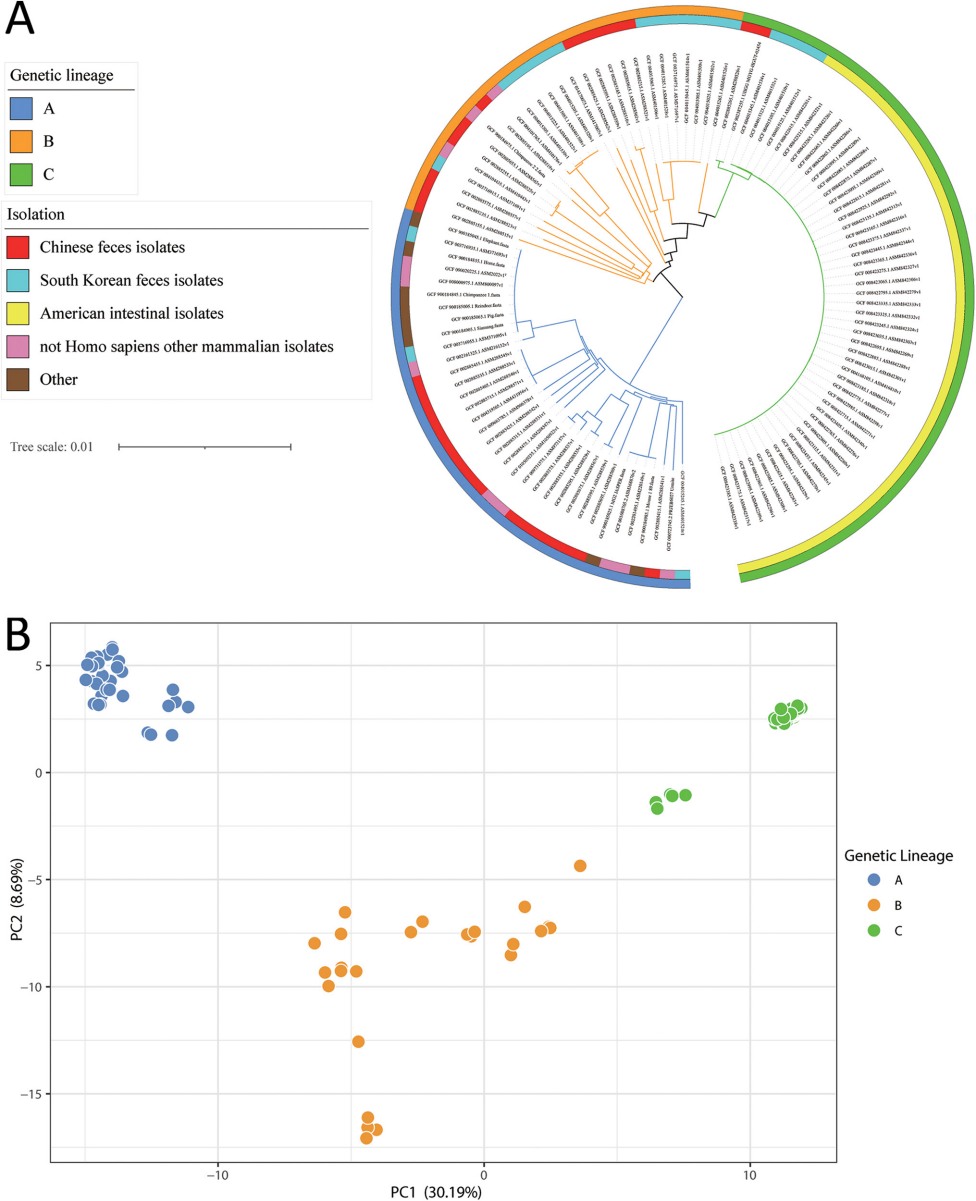
(A) A phylogenetic tree was constructed from 1,132 core genes of 112 Akkermansia muciniphila strains (average nucleotide identity [ANI] shared with the type strain BAA-835^T^ of >95%) in which the outer circle represents grouping of genetic lineages, and the inner circle represents characteristics of isolated sources. (B) Three genetic lineages were given different colors (A, blue; B, orange; C, green) based on principal-component analysis (PCA) based on pan-genome presence and absence matrices of 112 Akkermansia muciniphila strains. PC1 and PC2 represent the first and second principal components of principal component analysis respectively.

Based on the 1,132 core genes, we constructed a phylogenetic tree of the strains. The results of phylogenetic analysis showed that the 112 *A. muciniphila* strains are divided into three genetic lineages (A to C) by two main branches in the phylogenetic tree (Fig. 1A). The results of principal-component analysis based on the existence of pan-genes in the deletion matrix were consistent with the results of phylogenetic analysis, and all 112 *A. muciniphila* strains could be divided into three different genetic lineages at intraspecies level (Fig. 1B). It is worth mentioning that the *A. muciniphila* strains showed obvious regional aggregation in all three genetic lineages. Genetic lineage A was mainly composed of Chinese human fecal isolates (16/35, 45.71%) and not of mammalian isolates other than Homo sapiens (8/35, 22.86%). South Korean (13/27, 48.15%) and Chinese human fecal isolates (11/27, 40.74%) were the main components of genetic lineage B. Forty-four American (88.00%), four Korean (8.00%), and two Chinese intestinal isolates (4.00%) constituted genetic lineage C. In addition, genetic lineages B and C are parallel lineage groups which may share an ancestor, but both of them are distantly related to genetic lineage A.

### Average nucleic acid identity and total nucleic acid identity of *Akkermansia muciniphila*.

**FIG 2:**
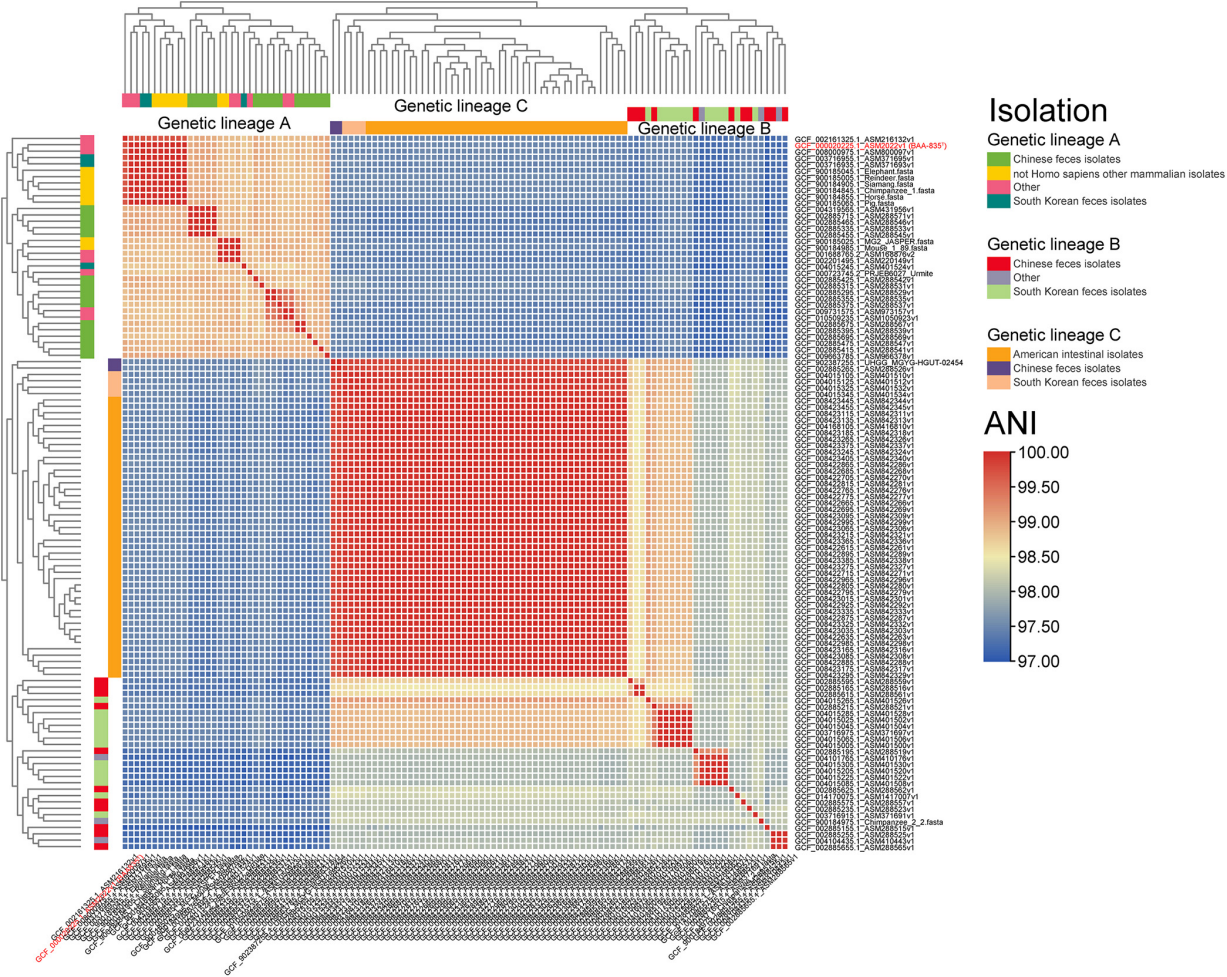
Heat map of average nucleotide identity results for pairs of 112 Akkermansia muciniphila strains, which were divided into three genetic lineages according to phylogenetic tree and principal-component analysis based on pan-genomic presence and absence matrices. Different colors represent the isolation characteristics of the three genetic lineages (genetic lineages A to C). The type strain BAA-835^T^ is marked in red for easy observation.

In order to analyze the genetic diversity and differences of different *A. muciniphila* genetic lineages, we used ANI and total nucleotide identity (TNI) to describe the genetic diversity and differences of the three genetic lineages. The ANI and TNI results (Fig. 2 and Fig. S3) were consistent with the results of the phylogenetic tree based on core gene and principal-component analysis (PCA) based on the presence of pan-gene deletion, and the 112 *A. muciniphila* strains could be divided into 3 genetic lineages at the species level. Genetic lineage A showed a significant difference with genetic lineages B and C (ANI < 98%, TNI < 92%). In contrast, genetic lineages B and C are relatively closely related (ANI > 98%). It is worth noting that genetic lineage C showed low genetic diversity in both ANI and TNI results (that is, different strains in the same lineage were highly related, leading to small genetic differences, ANI > 99.50%, TNI > 95%).

### Genetic characteristic differences between different genetic lineage strains of *Akkermansia muciniphila*.

**FIG 3:**
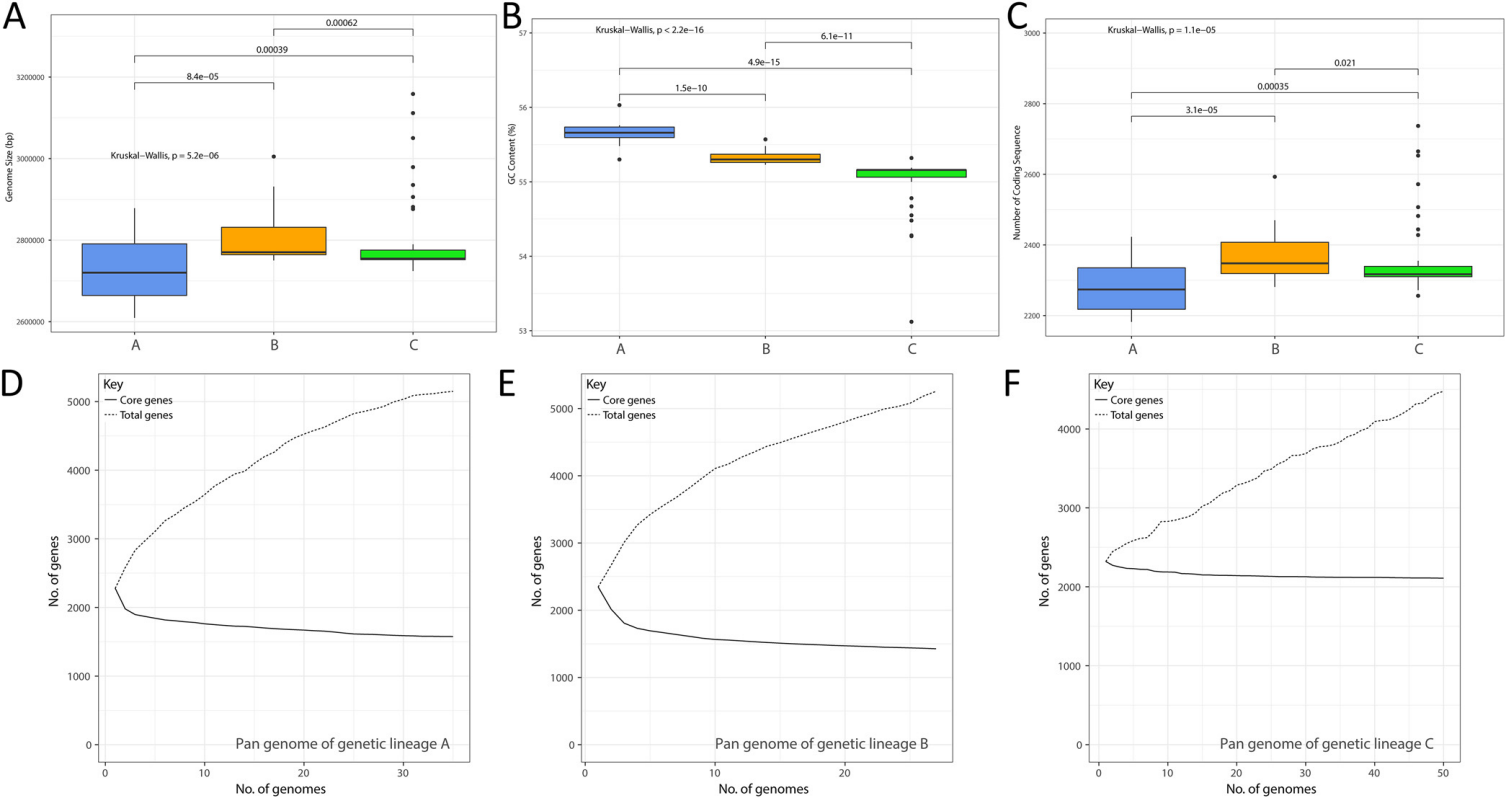
Differences in main genomic characteristics, (A) genome size, (B) GC content, and (C) number of coding genes, between the three genetic lineages of Akkermansia muciniphila (genetic lineages A to C). Pan-genomic and core gene sets of the three genetic lineages of Akkermansia muciniphila (genetic lineages A to C: panels D to F, respectively).

In order to analyze the genetic characteristic differences of different *A. muciniphila* genetic lineages, we compared their genome sizes, GC content, and numbers of CDS. The results showed that genome size (Fig. 3A), GC content (Fig. 3B), and the number of CDS (Fig. 3C) were significantly different between different genetic lineages (*P* = 5.2 × e^−6^, *P* < 2.2 × e^−16^, *P* = 1.1 × e^−5^; Kruskal-Wallis test). Genetic lineage A had the smallest genome and the lowest number of CDS, but the highest GC content. Genetic lineage C had the largest genome, the greatest number of coding sequences, and medium GC content. This is consistent with the good linear relationship between GC content and genome size found in this study.

Furthermore, we constructed pan-core gene sets of the three different genetic lineages. Genetic lineage A has 5,149 pan-genome genes and 1,576 core genome genes (Fig. 3D). Genetic lineage B has 5,255 pan genome genes and 1,427 core genome genes which form the core pan-gene set (Fig. 3E). Although lineage A has more strains than lineage B, the two lineages have about the same amounts of core genes and generic genes, suggesting that lineage B has fewer strains but higher genetic diversity. In contrast, lineage C has the largest number of strains of the three lineages (Fig. 3F) but the smallest pan-gene set (4,476 genes) and the largest core gene set (2,110). It is worth noting that the number of core genes in genetic lineage C accounted for an unusually dominant proportion of the genome CDS of each strain. Strain in genetic lineage C have 2,356 ± 102 CDS and core genes accounted for 89.56% of the average CDS number, suggesting that genetic lineage C had low genetic diversity and that different strains had high direct genetic relatedness.

### Lineage-specific core genes of difference genetic lineages of *Akkermansia muciniphila* strains.

The results of pan-genomic analysis of 112 *A. muciniphila* strains showed that genetic lineages B and C shared the most genes, and that lineage C had few lineage-specific genes. The three lineages possessed different spectra of lineage-specific core genes. Lineage C was the least diverse lineage, containing the highest number of core genes (2 coding genes and 26 hypothetical proteins) among the three lineages. Two interesting core putative proteins detected among lineage C isolates were the methyltransferase paeR7IM (a recombinant DNA repair enzyme of DNA helicase RecD2), which was specific to this lineage, as well as the tyrosine recombinase XerD, which exhibited a higher number of copies in lineage C than in other genetic lineages. Notably, the isolates in genetic lineage C did not contain the *RecBC* gene. The presence and enrichment of these two enzymes in genetic lineage C might result in the unique recombination characteristics and relatively low genomic diversity within this lineage. Eighteen lineage A-specific genes were identified, including 1 peptidyl-lysine *N*-acetyltransferase (*yajB*) and 17 hypothetical proteins. *N*-epsilon-lysine acetyltransferase participates in protein acetylation (18). No specific core genes were found in genetic lineage B, consistent with the relative high diversity within this lineage.

### Recombination, genomic islands, prophages, and CRISPR-Cas systems and spacers in different genetic lineages of *Akkermansia muciniphila*.

**FIG 4:**
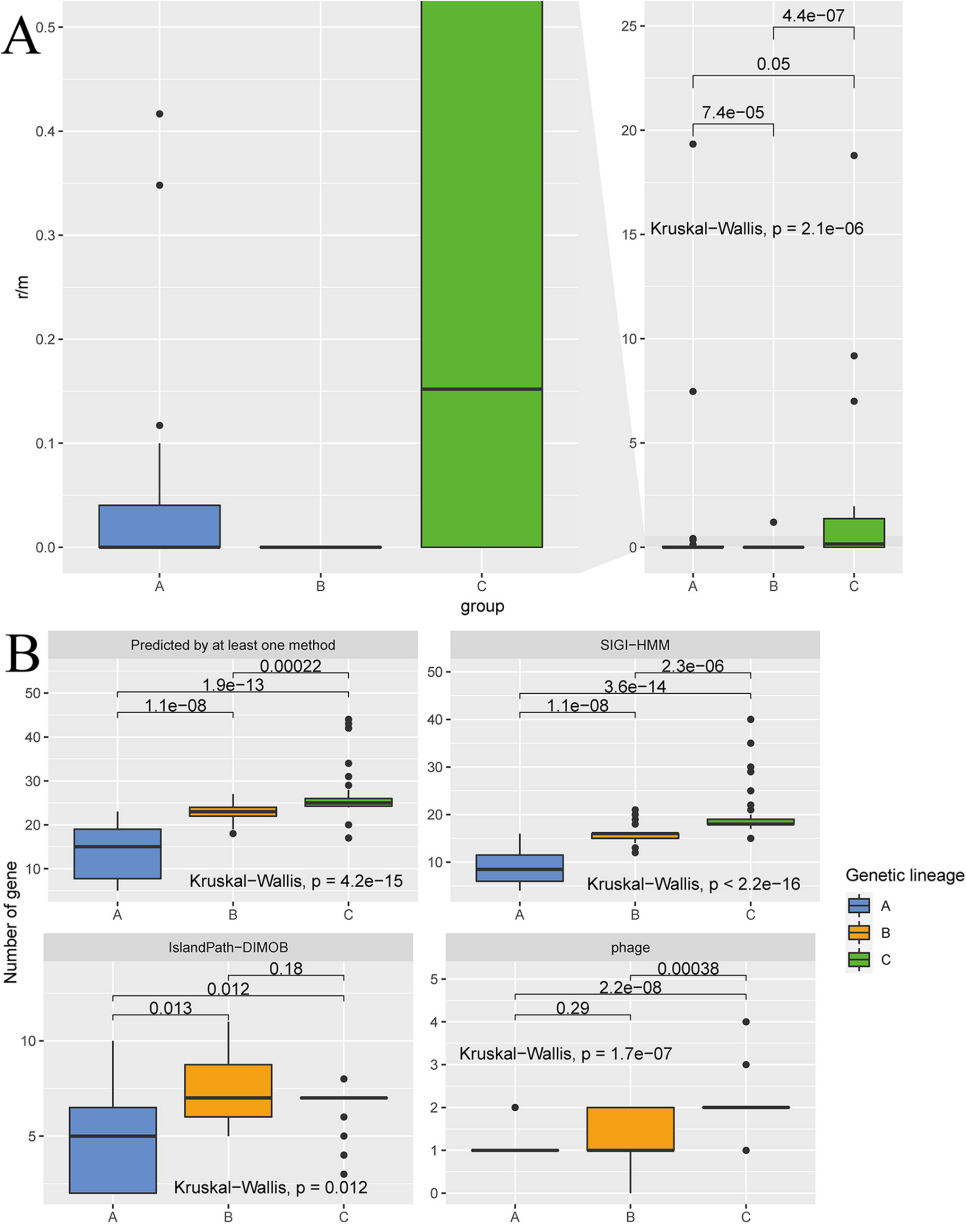
(A) Differences in recombination rates of 1,132 core genes among the three main genetic lineages of Akkermansia muciniphila (genetic lineages A to C). (B) Gene copy number differences in genomic islands (IslandViewer 4 software provided 3 methods of predicting genomic islands: predicted by at least one method, SIGI-HMM, and IslandPath-DIMOB) and number of phages.

A single recombination event typically affects a single DNA sequence, introducing multiple sites of variation. Therefore, Guttman and Dykhuizen (19) proposed using the *r/m* value to measure the degree of reorganization, where *r* is the number of mutation sites introduced by recombination and *m* is the number of mutation sites introduced by spontaneous mutation. The recombination rates of 1,132 core genes of 112 *A. muciniphila* strains were calculated, and there were significant differences in *r/m* among the three genetic lineages (*P* = 2.1 × e^−6^, Kruskal-Wallis test). The *r/m* rates of most strains were less than 1, indicating that mutation was the main evolutionary driving force in *A. muciniphila* strains. The different ratios of different genetic lineages indicate that different genetic lineages experienced different evolutionary events. Genetic lineage C had the highest recombination rate among the three genetic lineages, while genetic lineage B exhibited a much lower recombination rate. The recombination rate in genetic lineage B was close to zero (Fig. 4A).

Horizontal gene transfer is an important mechanism of nonhomologous recombination and one of the main driving forces of bacterial evolution. The presence of phage/prophage-containing genomic islands is indicative of horizontal gene transfer. Thus, the number of genomic islands and phages in the genomes of different genetic lineages were analyzed. The results showed that genetic lineage C had a significantly higher number of genomic islands (predicted by at least one method, *P* = 4.2 × e^−15^, Kruskal-Wallis test) and phages (*P* = 2.2 × e^−16^, Kruskal-Wallis test) (Fig. 4B). In contrast, genetic lineage A has the lowest number of genomic islands and prophages. In addition, we found that phages/prophages-containing genomic islands were observed in three different genetic lineages, indicating the existence of horizontal gene transfer (Fig. S4). Additionally, genetic lineages B and C commonly carry 3 copies of the SPBc2 prophage-derived glycosyltransferase *SunS* in phages/prophages-containing genomic islands (Fig. S4). These results suggested that different numbers and frequencies of horizontal gene transfer events occurred in the three lineages, and, generally, more of these events happened for the genetic lineage C isolates. Interestingly, *RecD2* and more copies of *XerD* were found on genomic islands in genetic lineage C, suggesting that genomic islands confer genes which are specific to genetic lineages and aid in lineage evolution.

In addition, we investigated the distribution of the CRISPR-Cas system across the three lineages. Generally, *A. muciniphila* could be divided into four groups according to the distribution and types of CRISPR-Cas systems present in their genomes, including no CRISPR-Cas, Type I-C, Type II-C, and Type I-C and II-C (Fig. S5A) (17). In genetic lineage A, 34.29% of isolates did not carry a CRISPR-Cas system, 20% had a Type I-C CRISPR-Cas system, 28.57% had a Type II-C CRISPR-Cas system, and 17.14% had Type I-C and II-C CRISPR-Cas systems (Fig. S5A). Genetic lineage B was dominated by CRISPR-Cas systems of Type I-C (40.74% of isolates) and Type II-C (29.63%). The Type I-C type was dominant in genetic lineage C.

Meanwhile, there was a significant difference in the number of CRISPR-Cas system spacers among *A. muciniphila* isolates of different genetic lineages (*P* = 1.2 × e^−16^, Kruskal-Wallis test; Fig. S5B). The isolates in genetic lineages C had the highest number of spaces (100.1 ± 19.3), followed by genetic lineages B (55.0 ± 27.9) and A (22.8 ± 27.5), respectively.

### Carbohydrate-active enzymes in *Akkermansia muciniphila*.

**FIG 5:**
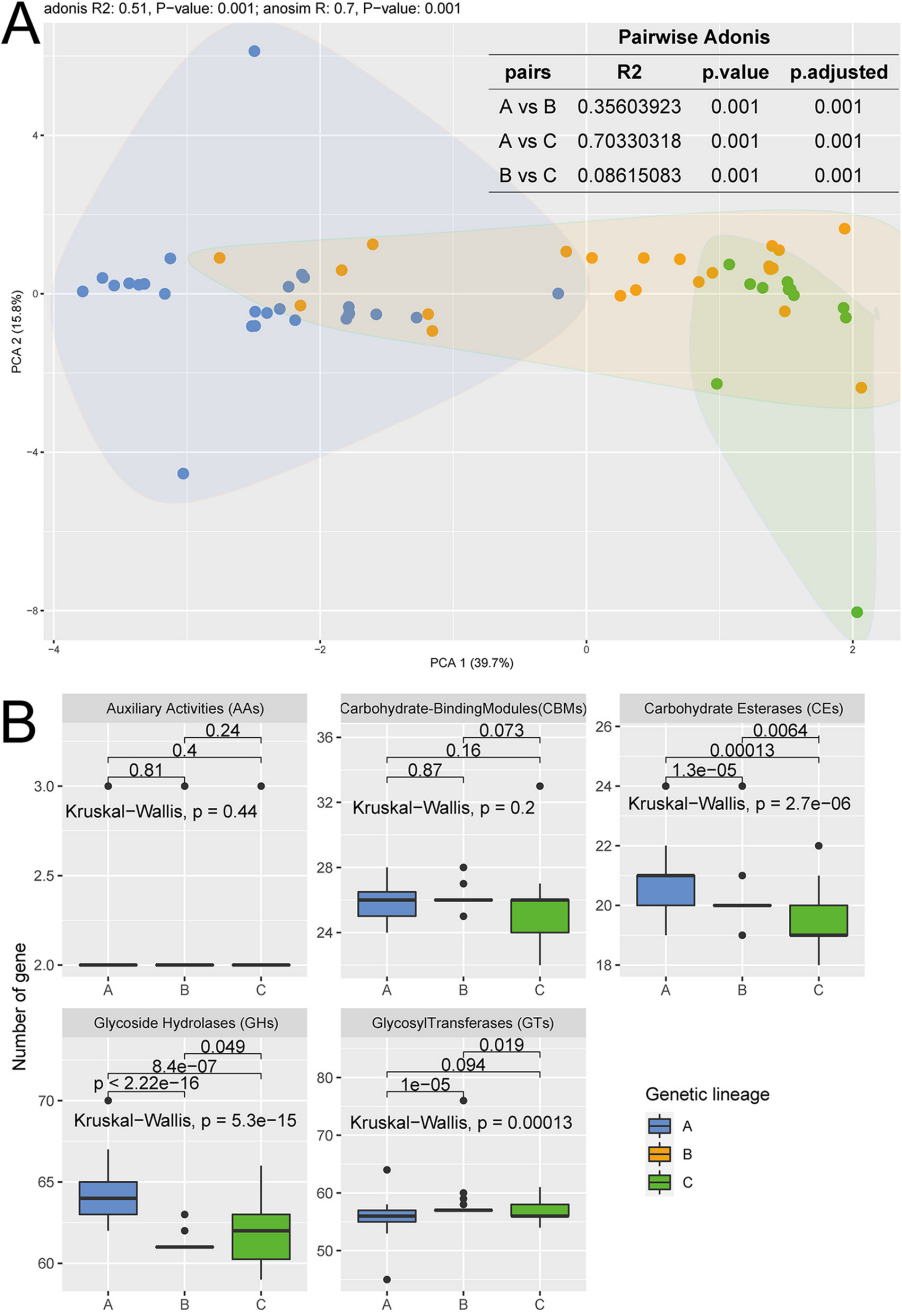
(A) Based on pairwise and non-pairwise adonis analysis (PERMANOVA), principal-component analysis was performed based on three genetic lineages using copy number matrices of carbohydrate related metabolism genes. Both adonis analysis and ANOSIM analysis showed significant differences (adonis: R^2^ = 0.51, *P* value = 0.001; anosim: R = 0.7, *P* value = 0.001); results of pairwise adonis analysis are shown in the table below. (B) Differences of five modules which catalyze the breakdown, biosynthesis, or modification of carbohydrates and glycoconjugates between the three genetic lineages of Akkermansia muciniphila (genetic lineages A to C). *P* values (Kruskal-Wallis test and Wilcoxon test) were obtained using R software (panel B).

The abundance of *A. muciniphila* in the gut can be increased through the intake of cranberry extract (20) or FODMAP (fermentable oligosaccharides, disaccharides, monosaccharides, and polyols) foods (21), suggesting great potential for dietary strategies in promoting the growth of intestinal *A. muciniphila*. Carbohydrate-active enzymes (CAZymes) play key roles in energy acquisition and utilization and growth in bacteria. Thus, the genomic profiles of *A. muciniphila* from different genetic lineages were compared. The results of PCA and permutational multivariate analysis of variance (PERMANOVA) revealed significant differences in the profiles of CAZyme-encoding genes between the three different genetic lineages (Fig. 5A; adonis, R^2^ = 0.51, *P* = 0.001; ANOSIM, R = 0.7, *P* = 0.001). Particularly, significant difference was observed between genetic lineage A and genetic lineages B and C based on pairwise adonis analysis (R^2^ = 0.36, *P* = 0.001; R^2^ = 0.70, *P* = 0.001).

Further analysis revealed significantly different makeup of CAZymes between the three separate lineages. Lineage A had a significantly higher number of glucoside hydrolases (GH family, *P* = 5.3 × e^−15^, Kruskal-Wallis test) and carbohydrate esterases (CE family, *P* = 2.7 × e^−6^, Kruskal-Wallis test) but a significantly lower number of glycosyl transferases (GT family, *P* = 0.00013, Kruskal-Wallis test) compared with lineages B and C. These results indicate that carbohydrate metabolism capacity varied between genetic lineages. Then, significant interlineage differences in the genomic contents of CAZymes were analyzed on a finer level. The results showed that the number of copies of CAZyme-encoding genes also varied between genetic lineages. Isolates in genetic lineage A had more copies of genes in the *GH33*, *GH43_24*, and *GT11* families (Fig. S6, *P* = 1.3 × e^−11^, *P* < 2.2 × e^−16^, *P* = 2.2 × e^−13,^ Kruskal-Wallis test), while genetic lineage B had fewer genes in the *CE6* family (4.06 ± 0.23 and 4.02 ± 0.14 copies of the *CE6* gene in genetic lineages A and C; 3.59 ± 0.56 copies of the *CE6* gene in genetic lineage B; *P* = 2.2 × e^−7^, Kruskal-Wallis test). In addition, the *GH18*, *CBM78*, and *GH141* gene families were unique to genetic lineage A, while the carbohydrate metabolite families of CBM56, GT5, and GT101 were common to genetic lineages B and C but not present in lineage A. These results together suggested that there were significant differences in carbohydrate metabolism capacity between the genetic lineages of *A. muciniphila*. The different carbohydrate metabolism capacities between genetic lineages and isolates could be related to their niches and the environmental selection forces that drive adaptation, particularly the dietary habits of the host, as *A. muciniphila* were mainly isolated the from mammalian gut.

## DISCUSSION

We retrieved 130 genome sequences of *A. muciniphila* from the NCBI RefSeq database, and pairwise ANI analysis confirmed that only 112 of these genomes fulfilled the requirement of 95% average nucleotide similarity when cross-compared with the type strain ATCC BAA-835^T^ (22). These 112 genomes were included in further bioinformatic analysis. The results of phylogenetic analysis showed that these isolates were clustered into three clades, and genetic lineage A was distinctive from genetic lineages B and C. Obvious region-based clustering patterns and interesting lineage-specific genomic features were observed. Specifically, one novel finding of this study was that the *XerD* and *RecD2* genes were mainly present in genetic lineage C and not present, or at least were far less frequent, in genetic lineages A and B; these genes were correlated with the recombination/mutation rate, the number of phages and genomic islands, the number and types of CRISPR-Cas systems, the pan-core genome set, and genetic diversity. Another novel observation was the presence of lineage-specific carbohydrate metabolism-related genes, suggesting different carbohydrate requirements and utilization capacities between *A. muciniphila* clades.

The *RecD2* gene was specific to lineage C, possibly leading to its distinctive pan-core genome set, lower genetic diversity, and higher recombination/mutation rate in the lineage core gene compared with lineages A and B. The RecD family consists of two helicases, RecD1 and RecD2. A previous phylogenetic study found clear segregation of these two proteins (23). RecD1 is a protein which systematically associates with RecBC to constitute the RecBCD helicase complex, while *RecD2* is found only in genomes which lack *RecBC* (23). Consistently, only the *RecD2* gene, not *RecBC*, was detected in *A. muciniphila* genomes. *RecD2* plays a key role in DNA repair (24, 25), and it also protected some bacteria from extremely harsh environments (26). Disrupting the *RecD2* gene in Bacillus anthracis resulted in a strong spontaneous mutator phenotype (27). The exact role of the *RecD2* gene in genetic lineage C of *A. muciniphila* is uncertain, and the reason for the distinctive distribution of such genes between clades is also unclear, but its specificity to genetic lineage C and its correlation with the small genetic diversity and low mutation/recombination rates in the population core gene might implicate a function in maintaining genomic stability.

Another clade-specific gene enriched in lineage C was *XerD*, which might play a role in promoting horizontal gene transfer, evidenced by the significantly greater number of genomic islands and phages in genomes of lineage C than in those of other lineages. XerC and XerD are tyrosine recombinases (28) that facilitate chromosomal integration of phages or other exogenous gene components (29). The presence of multiple copies of XerCD recombinase genes, *dif* sequences, and exogenous gene components around the *dif* sequences in the genomes in genetic lineage C might imply extensive integration of phages and associated gene components into bacterial chromosomes via site-specific recombination by the action of XerCD/*dif* (30). A recent study showed that *XerD* could unload bacterial chromosomal complexes at the replication end, and such a mechanism is important in maintaining genome organization in all living organisms (31). Horizontal gene transfer is an important mechanism which drives bacterial evolution and in turn shapes the bacterial genome (32, 33). This process also helps the microbes adapt to environmental niches. The host genome could effectively acquire foreign gene fragments, including active and functional motifs, through mobile elements and phage-derived genomic islands (34–36). Current results suggest that significantly more horizontal gene transfer events occurred in isolates of genetic lineage C than in those of other lineages.

Research on glucose metabolism in *A. muciniphila* were mostly performed for the type strain ATCC BAA-835^T^; thus, the carbohydrate metabolism capacity of *A. muciniphila* isolates remains to be explored. An *in vitro* study showed that *A. muciniphila* could utilize multiple monosaccharide substrates, including fucose, galactose, and *N*-acetylglucosamine (37). Plovier et al. (38) successfully cultivated the type strain ATCC BAA-835^T^ in a synthetic medium in which viscosin was replaced by a combination of glucose, *N*-acetylglucosamine, peptone, and threonine. Although this work was an *in silico* study, we nevertheless presented genome-based evidence showing significantly different genomic profiles of carbohydrate metabolism, and thus sugar metabolic potential, between genetic lineages and isolates. For example, the CAZYmes of the GH141 (including α-l-fucosidase and xylanase), CBM78, and GH18 families (including endo β-*N*-acetylglucosidase, peptidoglycan hydrolase with endo-β-*N*-acetylglucosaminidase activity) were specific to isolates of genetic lineage A.

Our results provide important implications for further investigating biological and physiological characteristics of different *A. muciniphila* strains/isolates; in particular, specific culture conditions and methods should be tailored for laboratory cultivation of isolates of interest. Moreover, it is worth mentioning that *A. muciniphila* is recognized as a keystone species in the human microbiome and a potential next-generation probiotic. Because most previous physiological and functional studies of *A. muciniphila* have been established from the type strain BAA-835^T^, knowledge and understanding of this species is limited (17). It is worth mentioning that probiotics should be defined at the strain level rather than the species level. Our results showed that there were some differences among *A. muciniphila* strains of different genetic lineages. Although the type strain ATCC BAA-835^T^ showed good probiotic characteristics, this does not mean that all *A. muciniphila* strains are probiotic. Second, because *A. muciniphila* strains are mostly concentrated on type strain ATCC BAA-835^T^, due to the genetic differences between different lineages, it is necessary to carefully analyze the phenotypic results brought by type strain ATCC BAA-835^T^. Finally, there is obvious regional aggregation in the phylogenetic background of *A. muciniphila*, and it may be necessary to develop *A. muciniphila* probiotic strains adapted to different geographical populations in the future.

## CONCLUSION

By comparative genomics analyses, our study showed that the 112 *A. muciniphila* genomes analyzed belonged to three genetic lineages, clustered by geographic origin and characterized by obvious differences in their profiles of genetic lineage-specific genes due to genomic islands, including *RecD2* (an ATP-dependent *RecD*-like DNA helicase) and *xerD* (a tyrosine recombinase). These lineage-specific genes might be associated with genomic features, such as the number of phages and genomic islands, the pan-core genome, the recombination rate, and genetic diversity. The profiles of carbohydrate metabolism/utilization genes (particularly the glycolytic hydrolase and carbohydrate esterase families) also varied between genetic lineages, suggesting different carbohydrate metabolism potential/requirements. Our findings provide important and practical implications for future research on *A. muciniphila*, especially for laboratory isolation/cultivation of strains of interest, selection of candidate strains for functional studies, and design and application of *A. muciniphila*-containing synbiotic formulations.

## MATERIALS AND METHODS

### Data source.

In September 2020, 130 sequenced genomes that were reported to be *A. muciniphila* were retrieved from the NCBI RefSeq database (39). Pairwise ANI between the type strain ATCC BAA-835^T^ and the retrieved sequences was calculated, and 112 strains were confirmed to be *A. muciniphila* (with shared ANI greater than 95%) and were included in further analysis. The information of the downloaded genome sequences is presented in Table S1 in the supplemental material.

### Calculation of ANI and TNI, gene prediction, and pan-genome analysis.

The values of pairwise ANI (15) and TNI (40) were calculated using previously described methods. ANI and TNI were calculated by referring to methods reported in previous literature (41). To normalize genome quality and ensure consistency for bioinformatics analyses in this study, the genome content of all retrieved *A. muciniphila* genomes was predicted using Prokka (v1.11) (42) using the default software parameters. The pan- and core genome sets of *A. muciniphila* were generated using Roary software (43) with the paramemter “-e -mafft -cd 100”. Recombination of the *A. muciniphila* core genome set was analyzed by Gubbins software (44) with default parameters.

### Phylogenetic analysis.

TreeBest software was used to construct a phylogenetic tree of single-copy tandem sequences of core genes generated by Roary based on gene adjacencies (number of repeats = 1,000 times; http://treesoft.sourceforge.net/treebest.shtml).

### Functional annotation of active carbohydrate enzymes, genomic islands, prophages, and CRISPR-Cas systems.

Functional gene annotation was performed by a BLAST search against the CAZy databases (45) using hmmscan (46) with the parameters E value < 1 × e^–10^, identity > 70%, and coverage percentage > 70%. All genome sequences were uploaded to IslandViewer 4 (47) to identify possible genome island regions in each genome. Prophages were identified by uploading all genome sequences to Phaster (48). CRISPR-Cas systems in the genomes were searched by an online analysis software, CRISPR-Cas Finder (49), which detected CRISPR gene clusters, cas genes, CRISPR-Cas types and subtypes, and sequence spacers. The types and differences in CRISPR-CAS gene clusters between genetic lineages were analyzed.

### Statistical analyses and plots.

Statistical analyses were implemented via the R platform. Heatmaps were generated using the pheatmap package, and PCA was performed using the psych package and visualized using the ggplot2 package. ANOSIM and adonis analysis (999 permutations) of PERMANOVA were performed using the vegan package in R software. Pairwise Adonis analysis (999 permutations, p.adjust.m = “BH”) of PERMANOVA was performed using the pairwiseAdonis package in R software. *P* values (Kruskal-Wallis test and Wilcoxon test) were calculated using R software.

### Data availability.

All genomic data used in this study are stored in NCBI. Information on the genome sequences is presented in Table S1 in the supplemental material.
