# Supplementary material for: Comparative Genomics Revealed Wide Intra-Species Genetic Heterogeneity and Lineage-Specific Genes of Akkermansia muciniphila
Source: Microbiol Spectr. 2022 May 10;10(3):e02439-21. doi: 10.1128/spectrum.02439-21 (PMC9241678; doi:10.1128/spectrum.02439-21)
Supplement: SUPPLEMENTAL FILE 1 — Supplemental material. Download spectrum.02439-21-s001.pdf, PDF file, 3.1 MB [file spectrum.02439-21-s001.pdf]

Supplement Figure

Figure S1

Average nucleotide identity (ANI, sub-figure A) and phylogenetic tree based on core genomics (sub-figure B) of all 130 *Akkermansia muciniphila* strains in NCBI.

Figure S2

There was an obvious linear relationship between the number of coding genes and the genomic size (sub-figure A), GC content (sub-figure B) and genomic size (sub-figure C), the number of coding genes and GC content of *Akkermansia muciniphila* isolates.

Figure S3

112 *Akkermansia muciniphila* strains were analyzed by total nucleotide identity (TNI) pairing.

Figure S4

Heat maps of Phages/Prophages-containing Genomic Islands predicted by methods Predicted by at least one method (sub-figure A), IslandPath-DIMOB (sub-figure B), and SIGI-HMM (sub-figure C). The colors of the heat map represent the gene copy number, and the groups represent the genetic lineage of the *Akkermansia muciniphila* strain

Figure S5

Types of CRISPR-CAS systems in *Akkermansia muciniphila*'s three genetic lineages (sub-figure A) and the number of spacers (sub-figure B).

Figure S6

Differences in carbohydrate metabolism enzymes of *Akkermansia muciniphila* strains of three genetic lineages.

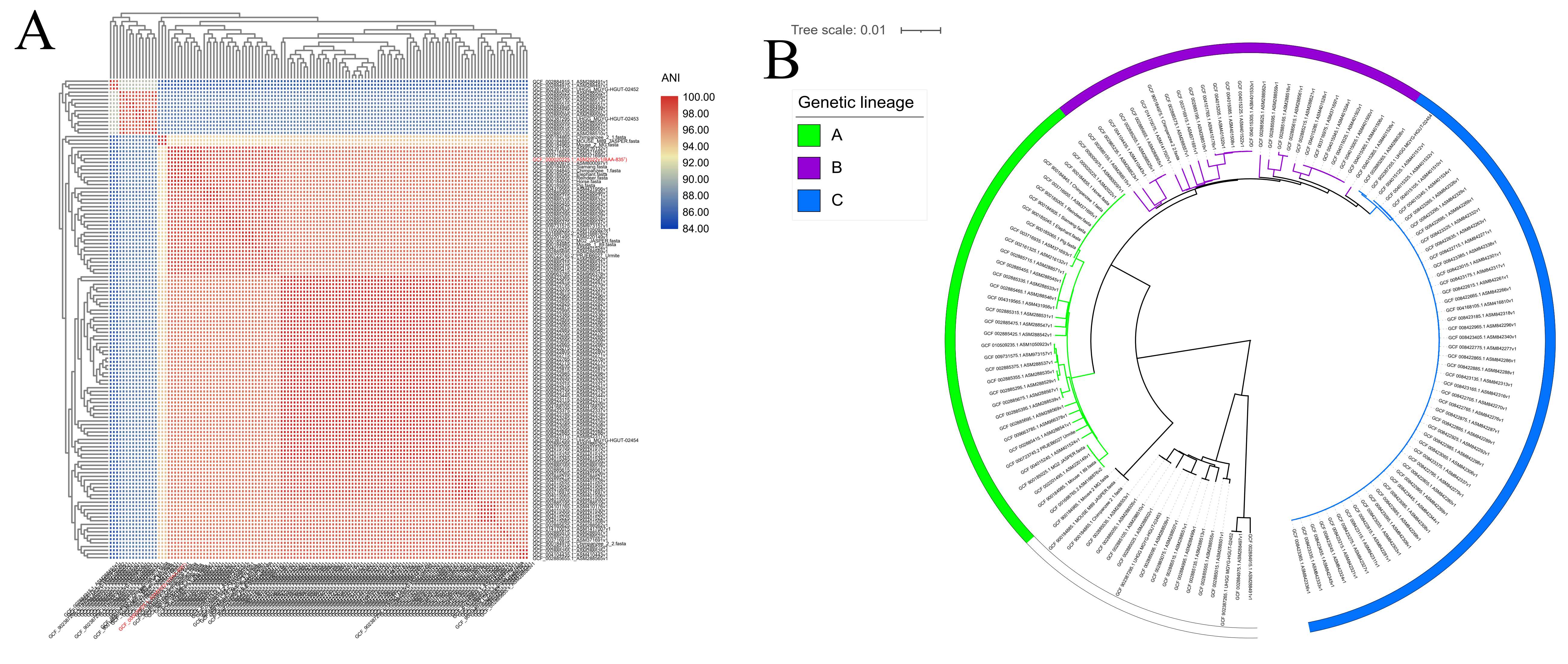
A
ANI
100.00
98.00
96.00
94.00
92.00
90.00
88.00
86.00
84.00
B
Tree scale: 0.01
Genetic lineage
A
B
C

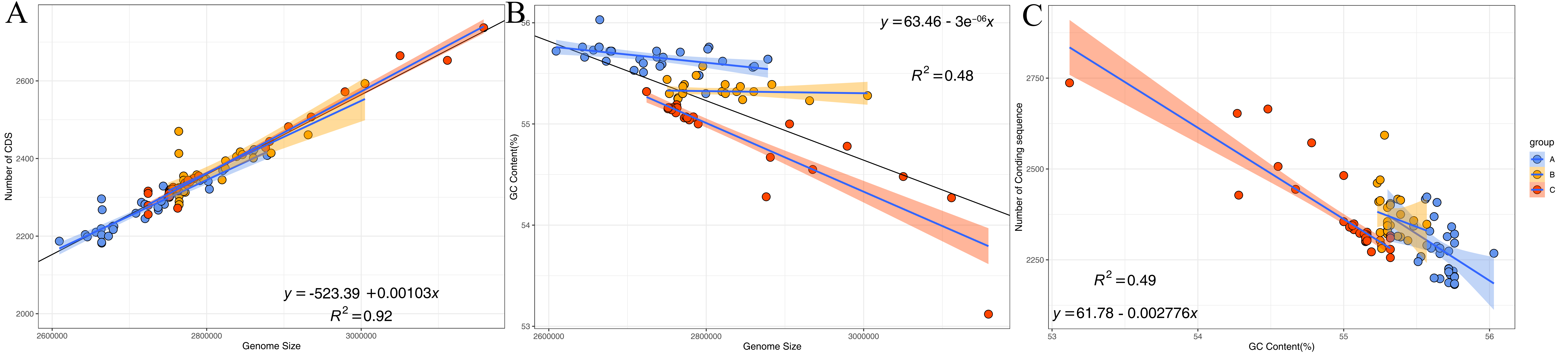

A
B
C
y = -523.39 +0.00103x
R^2 = 0.92
y = 63.46 - 3e^{-06}x
R^2 = 0.48
R^2 = 0.49
y = 61.78 - 0.002776x
Number of CDS
Genome Size
GC Content(%)
Genome Size
Number of Conding sequence
GC Content(%)
group
A
B
C

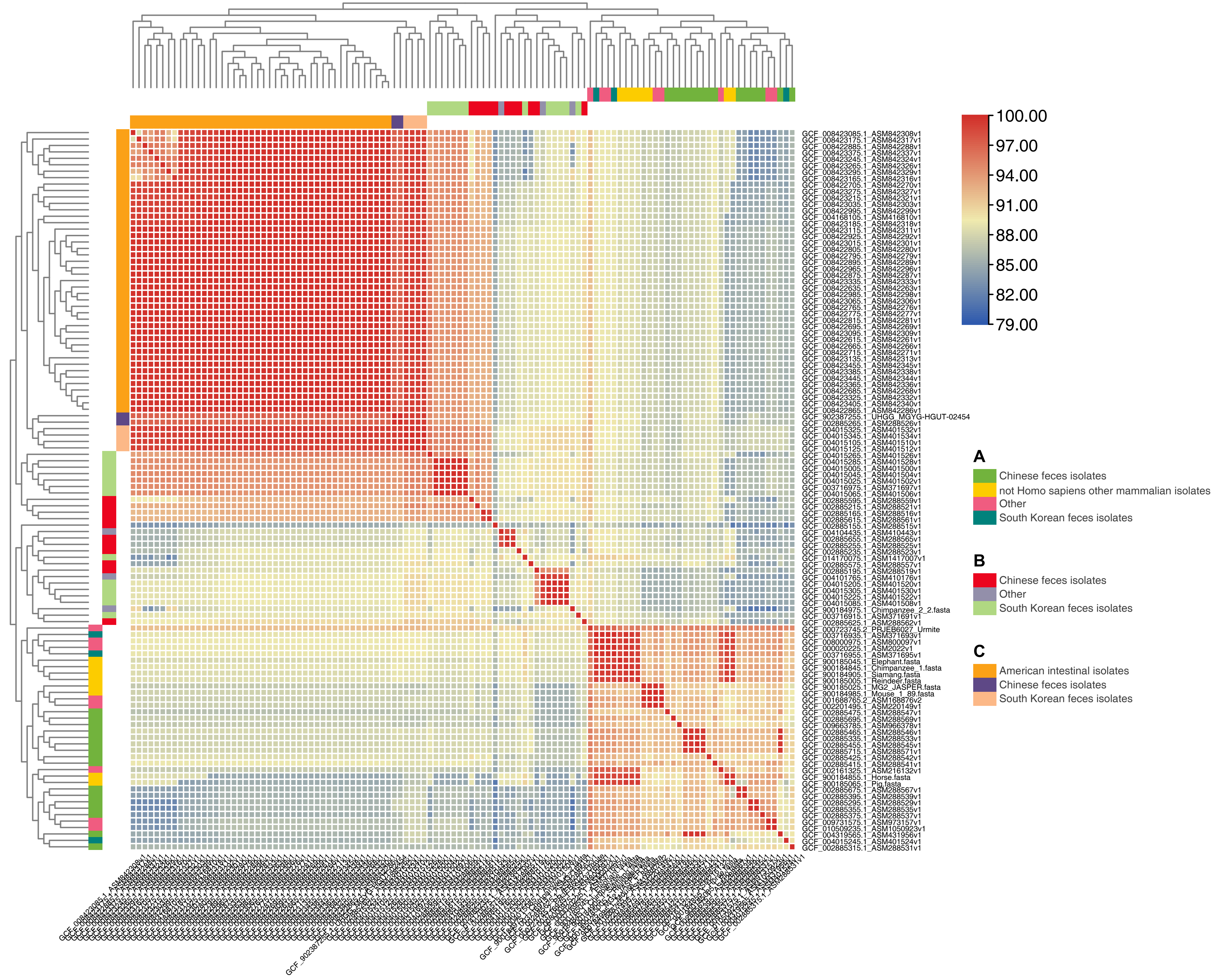

100.00
97.00
94.00
91.00
88.00
85.00
82.00
79.00
A
Chinese feces isolates
not Homo sapiens other mammalian isolates
Other
South Korean feces isolates
B
Chinese feces isolates
Other
South Korean feces isolates
C
American intestinal isolates
Chinese feces isolates
South Korean feces isolates

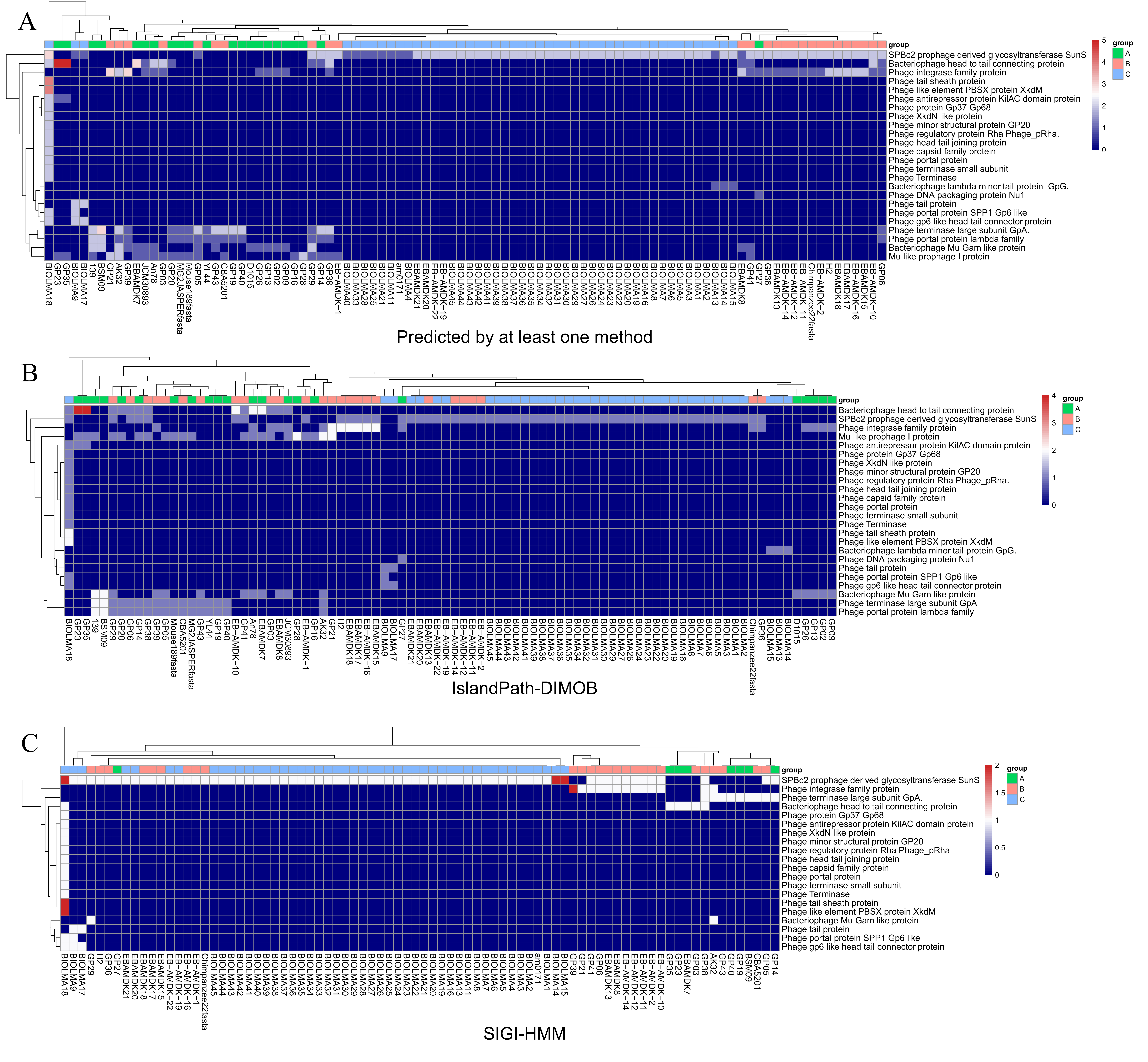
A
group
A
B
C
SPBc2 prophage derived glycosyltransferase SunS
Bacteriophage head to tail connecting protein
Phage integrase family protein
Phage tail sheath protein
Phage like element PBSX protein XkdM
Phage antirepressor protein KilAC domain protein
Phage protein Gp37 Gp68
Phage XkdN like protein
Phage minor structural protein GP20
Phage regulatory protein Rha Phage_pRha.
Phage head tail joining protein
Phage capsid family protein
Phage portal protein
Phage terminase small subunit
Phage Terminase
Bacteriophage lambda minor tail protein GpG.
Phage DNA packaging protein Nu1
Phage tail protein
Phage portal protein SPP1 Gp6 like
Phage gp6 like head tail connector protein
Phage terminase large subunit GpA.
Phage portal protein lambda family
Bacteriophage Mu Gam like protein
Mu like prophage I protein
Predicted by at least one method
B
group
A
B
C
Bacteriophage head to tail connecting protein
SPBc2 prophage derived glycosyltransferase SunS
Phage integrase family protein
Mu like prophage I protein
Phage antirepressor protein KilAC domain protein
Phage protein Gp37 Gp68
Phage XkdN like protein
Phage minor structural protein GP20
Phage regulatory protein Rha Phage_pRha.
Phage head tail joining protein
Phage capsid family protein
Phage portal protein
Phage terminase small subunit
Phage Terminase
Phage tail sheath protein
Phage like element PBSX protein XkdM
Bacteriophage lambda minor tail protein GpG.
Phage DNA packaging protein Nu1
Phage tail protein
Phage portal protein SPP1 Gp6 like
Phage gp6 like head tail connector protein
Bacteriophage Mu Gam like protein
Phage terminase large subunit GpA
Phage portal protein lambda family
IslandPath-DIMOB
C
group
A
B
C
SPBc2 prophage derived glycosyltransferase SunS
Phage integrase family protein
Phage terminase large subunit GpA.
Bacteriophage head to tail connecting protein
Phage protein Gp37 Gp68
Phage antirepressor protein KilAC domain protein
Phage XkdN like protein
Phage minor structural protein GP20
Phage regulatory protein Rha Phage_pRha
Phage head tail joining protein
Phage capsid family protein
Phage portal protein
Phage terminase small subunit
Phage Terminase
Phage tail sheath protein
Phage like element PBSX protein XkdM
Bacteriophage Mu Gam like protein
Phage tail protein
Phage portal protein SPP1 Gp6 like
Phage gp6 like head tail connector protein
SIGI-HMM

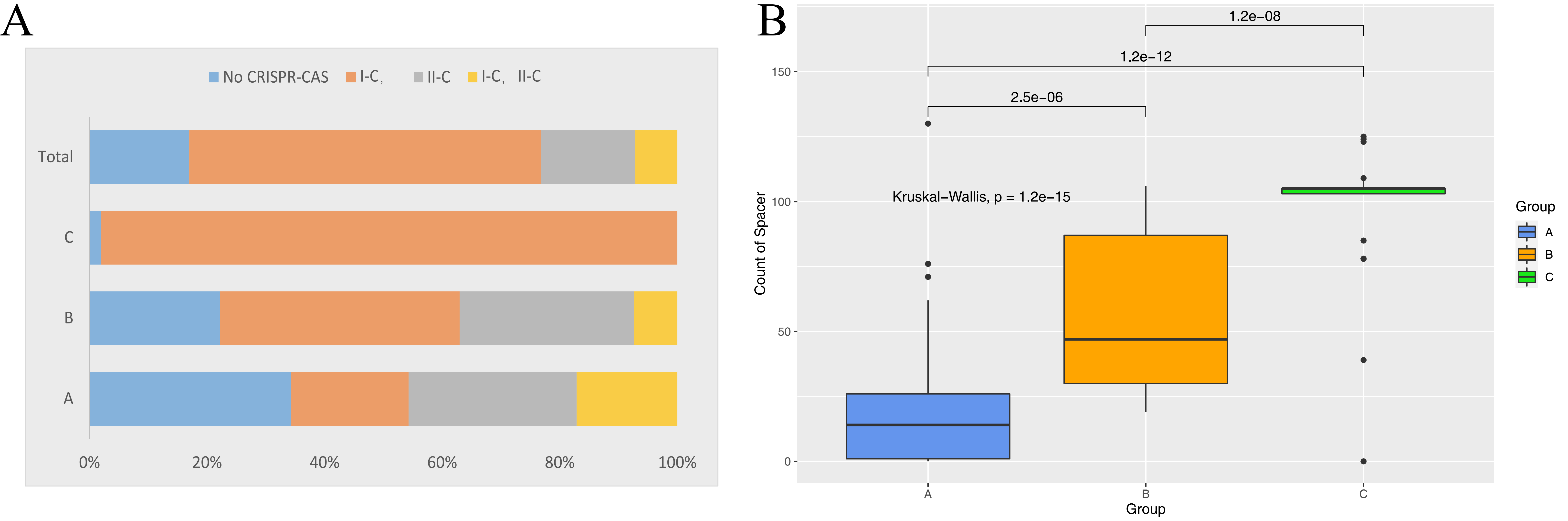

A
No CRISPR-CAS
I-C,
II-C
I-C, II-C
Total
C
B
A
0%
20%
40%
60%
80%
100%
B
1.2e−08
1.2e−12
2.5e−06
Kruskal−Wallis, p = 1.2e−15
Count of Spacer
150
100
50
0
A
B
C
Group
Group
A
B
C

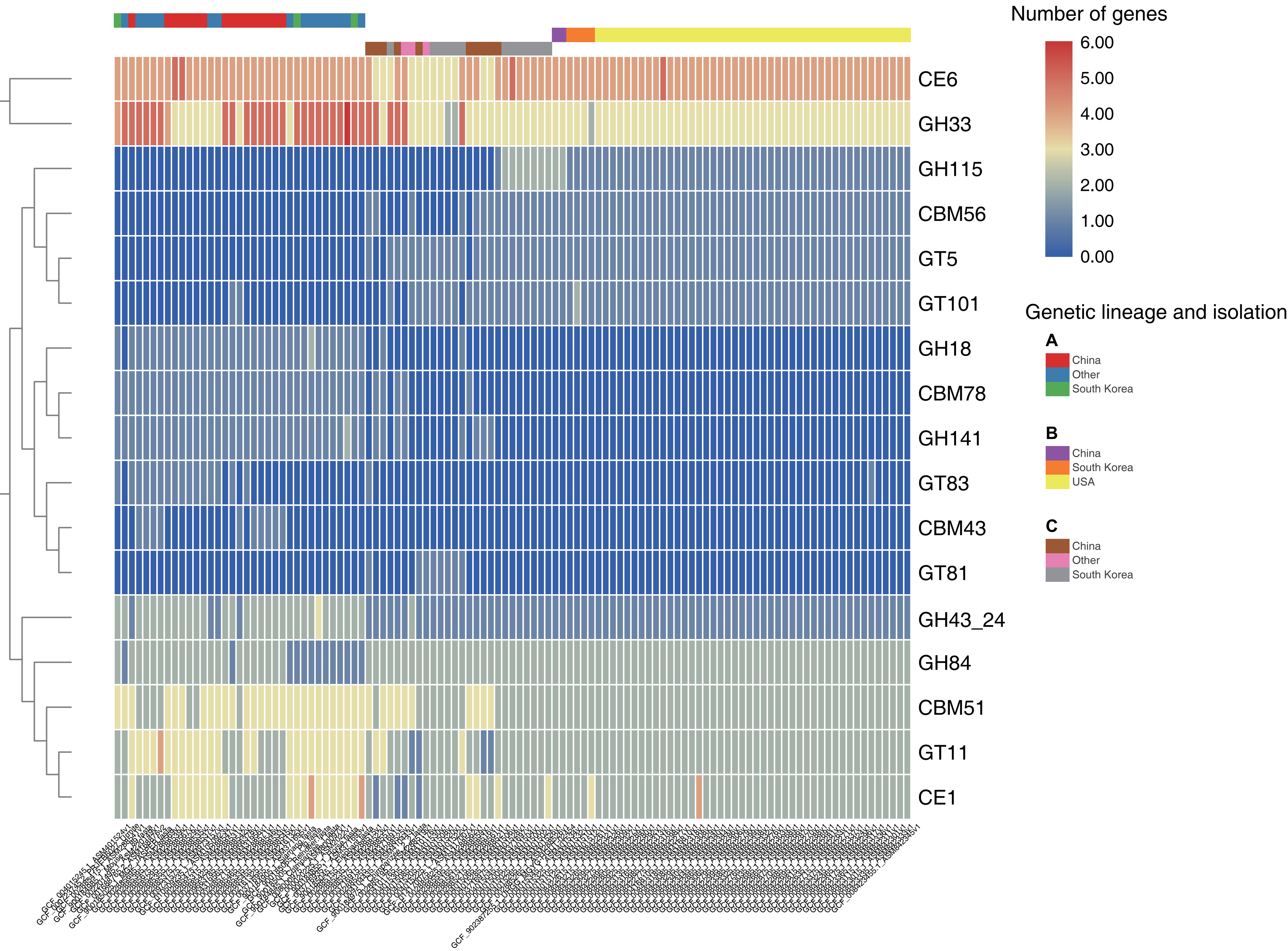
Number of genes
6.00
5.00
4.00
3.00
2.00
1.00
0.00
Genetic lineage and isolation
A
China
Other
South Korea
B
China
South Korea
USA
C
China
Other
South Korea
CE6
GH33
GH115
CBM56
GT5
GT101
GH18
CBM78
GH141
GT83
CBM43
GT81
GH43_24
GH84
CBM51
GT11
CE1

Table S1

	Strain	BioSample	BioProject	Assembly	Level	Size(Mb)	GC%	WGS	Scaffolds	CDS	Release Date
1	ATCC BAA-835	SAMN00138213	PRJNA20089	GCA_000020225.1	●	2.66	55.8		1	2122	5-May-08
2	Urmite	SAMEA3139045	PRJEB6027	GCA_000723745.2	◑	2.66	55.7	CCDQ01	1	2038	16-May-14
3	YL44	SAMN04621615	PRJNA317592	GCA_001688765.2	●	2.75	55.7		1	2239	20-Jul-16
4	An78	SAMN06473757	PRJNA377666	GCA_002161325.1	◔	2.74	55.7	NFHJ01	10	2219	30-May-17
5	YL44	SAMN03854119	PRJNA289613	GCA_002201495.1	◕	2.74	55.7		1	2218	20-Jun-17
6	GP24	SAMN08162557	PRJNA331216	GCA_002884915.1	◑	3.01	58	PJKA01	14	2426	16-Jan-18
7	GP22	SAMN08162556	PRJNA331216	GCA_002884975.1	◔	2.9	58.3	PJKB01	7	2315	16-Jan-18
8	GP30	SAMN08162554	PRJNA331216	GCA_002884995.1	◑	3.19	57.8	PJKD01	21	2591	16-Jan-18
9	GP12	SAMN08162551	PRJNA331216	GCA_002885015.1	◑	3.11	57.8	PJKG01	20	2560	16-Jan-18
10	GP15	SAMN08162552	PRJNA331216	GCA_002885025.1	◔	3.17	57.9	PJKF01	21	2579	16-Jan-18
11	GP11	SAMN08162550	PRJNA331216	GCA_002885055.1	◔	3.17	57.7	PJKH01	42	2596	16-Jan-18
12	GP08	SAMN08162548	PRJNA331216	GCA_002885075.1	◔	3.1	58.1	PJKJ01	27	2513	16-Jan-18
13	GP07	SAMN08162547	PRJNA331216	GCA_002885095.1	◔	3.1	58.1	PJKK01	15	2512	16-Jan-18
14	BSH05	SAMN08162546	PRJNA331216	GCA_002885105.1	◑	2.7	57.8	PJKL01	23	2205	16-Jan-18
15	BSH01	SAMN08162545	PRJNA331216	GCA_002885135.1	◑	2.66	58.1	PJKM01	16	2131	16-Jan-18
16	GP43	SAMN08162544	PRJNA331216	GCA_002885155.1	◑	2.8	55.6	PJKN01	11	2267	16-Jan-18
17	GP41	SAMN08162543	PRJNA331216	GCA_002885165.1	◔	2.77	55.4	PJKO01	14	2251	16-Jan-18
18	GP36	SAMN08162540	PRJNA331216	GCA_002885195.1	◔	2.75	55.4	PJKR01	11	2241	16-Jan-18
19	GP29	SAMN08162539	PRJNA331216	GCA_002885215.1	◑	2.75	55.3	PJKS01	20	2261	16-Jan-18
20	GP21	SAMN08162538	PRJNA331216	GCA_002885235.1	◔	2.84	55.4	PJKT01	18	2333	16-Jan-18
21	GP03	SAMN08162534	PRJNA331216	GCA_002885255.1	◑	2.82	55.3	PJKX01	19	2306	16-Jan-18
22	GP01	SAMN08162533	PRJNA331216	GCA_002885265.1	◔	2.76	55.2	PJKY01	1	2202	16-Jan-18
23	GP35	SAMN08162531	PRJNA331216	GCA_002885295.1	◑	2.86	55.6	PJLA01	14	2347	16-Jan-18

24	GP27	SAMN08162529	PRJNA331216	GCA_002885315.1		2.72	55.5	PJLC01	11	2189	16-Jan-18
25	GP26	SAMN08162528	PRJNA331216	GCA_002885335.1		2.68	55.7	PJLD01	17	2172	16-Jan-18
26	GP23	SAMN08162527	PRJNA331216	GCA_002885355.1		2.86	55.6	PJLE01	10	2336	16-Jan-18
27	GP20	SAMN08162526	PRJNA331216	GCA_002885375.1		2.82	55.6	PJLF01	15	2301	16-Jan-18
28	GP19	SAMN08162525	PRJNA331216	GCA_002885395.1		2.79	55.5	PJLG01	10	2280	16-Jan-18
29	GP16	SAMN08162524	PRJNA331216	GCA_002885415.1		2.67	55.6	PJLH01	13	2147	16-Jan-18
30	GP14	SAMN08162523	PRJNA331216	GCA_002885425.1		2.71	55.5	PJLI01	17	2200	16-Jan-18
31	GP13	SAMN08162522	PRJNA331216	GCA_002885455.1		2.68	55.7	PJLJ01	16	2182	16-Jan-18
32	GP02	SAMN08162520	PRJNA331216	GCA_002885465.1		2.68	55.7	PJLL01	20	2179	16-Jan-18
33	BSM09	SAMN08162519	PRJNA331216	GCA_002885475.1		2.65	55.7	PJLM01	18	2166	16-Jan-18
34	GP42	SAMN08162555	PRJNA331216	GCA_002885515.1		3.2	57.8	PJKC01	14	2597	16-Jan-18
35	GP25	SAMN08162553	PRJNA331216	GCA_002885535.1		3.14	57.8	PJKE01	23	2571	16-Jan-18
36	GP10	SAMN08162549	PRJNA331216	GCA_002885555.1		3.2	57.7	PJKI01	24	2667	16-Jan-18
37	GP39	SAMN08162542	PRJNA331216	GCA_002885575.1		2.88	55.4	PJKP01	23	2353	16-Jan-18
38	GP38	SAMN08162541	PRJNA331216	GCA_002885595.1		2.85	55.2	PJKQ01	18	2344	16-Jan-18
39	GP06	SAMN08162537	PRJNA331216	GCA_002885615.1		2.77	55.3	PJKU01	19	2264	16-Jan-18
40	GP05	SAMN08162536	PRJNA331216	GCA_002885625.1		2.79	55.5	PJKV01	11	2285	16-Jan-18
41	GP04	SAMN08162535	PRJNA331216	GCA_002885655.1		2.84	55.3	PJKW01	22	2328	16-Jan-18
42	GP40	SAMN08162532	PRJNA331216	GCA_002885675.1		2.79	55.5	PJKZ01	12	2271	16-Jan-18
43	GP28	SAMN08162530	PRJNA331216	GCA_002885695.1		2.72	55.6	PJLB01	13	2220	16-Jan-18
44	GP09	SAMN08162521	PRJNA331216	GCA_002885715.1		2.68	55.7	PJLK01	19	2183	16-Jan-18
45	UBA10767	SAMN08018620	PRJNA417962	GCA_003497705.1		1.63	55	DNOI01	336	1617	7-Sep-18
46	UBA8862	SAMN08019340	PRJNA417962	GCA_003514565.1		2.59	55.8	DOPV01	31	2221	7-Sep-18
47	EB-AMDK-1	SAMN07978491	PRJNA417186	GCA_003716915.1		2.77	55.4		1	2231	7-Nov-18
48	EB-AMDK-3	SAMN07980909	PRJNA417203	GCA_003716935.1		2.66	55.8		1	2123	7-Nov-18
49	EB-AMDK-4	SAMN07980958	PRJNA417208	GCA_003716955.1		2.66	55.8		1	2130	7-Nov-18

50	EB-AMDK-2	SAMN07980963	PRJNA417215	GCA_003716975.1	●	2.76	55.3		1	2235	7-Nov-18
51	EB-AMDK-10	SAMN08329147	PRJNA429085	GCA_004015005.1	●	2.76	55.2		1	2260	14-Jan-19
52	EB-AMDK-11	SAMN08329199	PRJNA429088	GCA_004015025.1	●	2.76	55.3		1	2224	14-Jan-19
53	EB-AMDK-12	SAMN08334970	PRJNA429277	GCA_004015045.1	●	2.76	55.3		1	2227	14-Jan-19
54	EB-AMDK-14	SAMN08334973	PRJNA429282	GCA_004015065.1	●	2.76	55.3		1	2240	14-Jan-19
55	EB-AMDK-18	SAMN08334978	PRJNA429288	GCA_004015085.1	●	2.77	55.3		1	2242	14-Jan-19
56	EB-AMDK-19	SAMN08334979	PRJNA429289	GCA_004015105.1	●	2.72	55.3		1	2159	14-Jan-19
57	EB-AMDK-22	SAMN08334983	PRJNA429295	GCA_004015125.1	●	2.72	55.3		1	2168	14-Jan-19
58	EB-AMDK-16	SAMN08334976	PRJNA429285	GCA_004015205.1	●	2.77	55.3		1	2231	14-Jan-19
59	EB-AMDK-17	SAMN08334977	PRJNA429286	GCA_004015225.1	●	2.77	55.3		1	2244	14-Jan-19
60	EB-AMDK-7	SAMN08328879	PRJNA429075	GCA_004015245.1	●	2.8	55.3		1	2279	14-Jan-19
61	EB-AMDK-8	SAMN08329008	PRJNA429077	GCA_004015265.1	●	2.82	55.4		1	2295	14-Jan-19
62	EB-AMDK-13	SAMN08334972	PRJNA429279	GCA_004015285.1	●	2.76	55.3		1	2247	14-Jan-19
63	EB-AMDK-15	SAMN08334975	PRJNA429284	GCA_004015305.1	●	2.77	55.3		1	2243	14-Jan-19
64	EB-AMDK-20	SAMN08334981	PRJNA429292	GCA_004015325.1	●	2.72	55.3		1	2177	14-Jan-19
65	EB-AMDK-21	SAMN08334982	PRJNA429294	GCA_004015345.1	●	2.72	55.3		1	2182	14-Jan-19
66	H2	SAMN03278370	PRJNA270907	GCA_004101765.1	◕	2.82	55.3		1	2264	22-Jan-19
67	CBA5201	SAMN10186677	PRJNA494944	GCA_004104435.1	●	2.86	55.3		1	2336	24-Jan-19
68	am_0171	SAMN10239579	PRJNA496358	GCA_004168105.1	◔	2.77	55.1	RCXB01	41	2238	11-Feb-19
69	139	SAMN10963682	PRJNA523108	GCA_004319565.1	●	2.8	55.7		1	2297	28-Feb-19
70	W0P28.008	SAMN10183248	PRJNA494875	GCA_004557455.1	◔	2.55	59.9	SFDX01	88		1-Apr-19
71	W0P25.047	SAMN10183247	PRJNA494875	GCA_004557465.1	◔	2.56	56.2	SFDV01	166		1-Apr-19
72	DSM 22959	SAMN12556229	PRJNA559704	GCA_008000975.1	●	2.66	55.8		1	2124	18-Aug-19
73	BIOML-A45	SAMN11943045	PRJNA544527	GCA_008422615.1	◑	2.75	55.2	VVKN01	17	2222	16-Sep-19
74	BIOML-A44	SAMN11943044	PRJNA544527	GCA_008422635.1	◑	2.75	55.2	VVKM01	22	2221	16-Sep-19
75	BIOML-A43	SAMN11943043	PRJNA544527	GCA_008422665.1	◑	2.75	55.2	VVKL01	20	2221	16-Sep-19

76	BIOML-A39	SAMN11943039	PRJNA544527	GCA_008422685.1		2.76	55.1	VVKH01	24	2228	16-Sep-19
77	BIOML-A41	SAMN11943041	PRJNA544527	GCA_008422695.1		2.75	55.2	VVKJ01	21	2225	16-Sep-19
78	BIOML-A40	SAMN11943040	PRJNA544527	GCA_008422705.1		2.79	55	VVKI01	35	2265	16-Sep-19
79	BIOML-A42	SAMN11943042	PRJNA544527	GCA_008422715.1		2.75	55.2	VVKK01	22	2225	16-Sep-19
80	BIOML-A36	SAMN11943036	PRJNA544527	GCA_008422765.1		2.75	55.1	VVKE01	26	2220	16-Sep-19
81	BIOML-A37	SAMN11943037	PRJNA544527	GCA_008422775.1		2.75	55.2	VVKF01	21	2224	16-Sep-19
82	BIOML-A35	SAMN11943035	PRJNA544527	GCA_008422795.1		2.75	55.2	VVKD01	23	2223	16-Sep-19
83	BIOML-A34	SAMN11943034	PRJNA544527	GCA_008422805.1		2.75	55.2	VVKC01	24	2220	16-Sep-19
84	BIOML-A38	SAMN11943038	PRJNA544527	GCA_008422815.1		2.75	55.2	VVKG01	21	2219	16-Sep-19
85	BIOML-A32	SAMN11943032	PRJNA544527	GCA_008422865.1		2.75	55.2	VVKA01	21	2223	16-Sep-19
86	BIOML-A30	SAMN11943030	PRJNA544527	GCA_008422875.1		2.75	55.2	VVJY01	23	2217	16-Sep-19
87	BIOML-A33	SAMN11943033	PRJNA544527	GCA_008422885.1		2.91	55	VVKB01	105	2390	16-Sep-19
88	BIOML-A31	SAMN11943031	PRJNA544527	GCA_008422895.1		2.75	55.2	VVJZ01	24	2227	16-Sep-19
89	BIOML-A29	SAMN11943029	PRJNA544527	GCA_008422925.1		2.75	55.2	VVJX01	25	2218	16-Sep-19
90	BIOML-A27	SAMN11943027	PRJNA544527	GCA_008422965.1		2.75	55.2	VVJV01	22	2215	16-Sep-19
91	BIOML-A26	SAMN11943026	PRJNA544527	GCA_008422985.1		2.75	55.2	VVJU01	22	2230	16-Sep-19
92	BIOML-A28	SAMN11943028	PRJNA544527	GCA_008422995.1		2.78	55.1	VVJW01	31	2248	16-Sep-19
93	BIOML-A24	SAMN11943024	PRJNA544527	GCA_008423015.1		2.75	55.2	VVJS01	23	2226	16-Sep-19
94	BIOML-A25	SAMN11943025	PRJNA544527	GCA_008423035.1		2.78	55	VVJT01	31	2253	16-Sep-19
95	BIOML-A23	SAMN11943023	PRJNA544527	GCA_008423065.1		2.75	55.2	VVJR01	22	2218	16-Sep-19
96	BIOML-A21	SAMN11943021	PRJNA544527	GCA_008423085.1		3.11	54.3	VVJP01	107	2530	16-Sep-19
97	BIOML-A22	SAMN11943022	PRJNA544527	GCA_008423095.1		2.75	55.2	VVJQ01	20	2222	16-Sep-19
98	BIOML-A20	SAMN11943020	PRJNA544527	GCA_008423115.1		2.75	55.2	VVJO01	25	2213	16-Sep-19
99	BIOML-A19	SAMN11943019	PRJNA544527	GCA_008423135.1		2.76	55.1	VVJN01	25	2249	16-Sep-19
100	BIOML-A17	SAMN11943017	PRJNA544527	GCA_008423165.1		3.05	54.5	VVJL01	237	2584	16-Sep-19
101	BIOML-A18	SAMN11943018	PRJNA544527	GCA_008423175.1		3.16	53.1	VVJM01	49	2647	16-Sep-19

102	BIOML-A16	SAMN11943016	PRJNA544527	GCA_008423185.1	◑	2.77	55.1	VVJK01	30	2238	16-Sep-19
103	BIOML-A15	SAMN11943015	PRJNA544527	GCA_008423215.1	◑	2.78	55.1	VVJJ01	46	2262	16-Sep-19
104	BIOML-A14	SAMN11943014	PRJNA544527	GCA_008423245.1	◑	2.88	54.3	VVJI01	82	2349	16-Sep-19
105	BIOML-A13	SAMN11943013	PRJNA544527	GCA_008423265.1	◑	2.88	54.7	VVJH01	77	2330	16-Sep-19
106	BIOML-A11	SAMN11943011	PRJNA544527	GCA_008423275.1	◑	2.78	55.1	VVJG01	32	2256	16-Sep-19
107	BIOML-A9	SAMN11943009	PRJNA544527	GCA_008423295.1	◑	2.98	54.8	VVJF01	121	2485	16-Sep-19
108	BIOML-A8	SAMN11943008	PRJNA544527	GCA_008423325.1	◑	2.76	55.1	VVJE01	26	2225	16-Sep-19
109	BIOML-A7	SAMN11943007	PRJNA544527	GCA_008423335.1	◑	2.75	55.2	VVJD01	24	2219	16-Sep-19
110	BIOML-A6	SAMN11943006	PRJNA544527	GCA_008423365.1	◑	2.76	55.2	VVJC01	18	2224	16-Sep-19
111	BIOML-A4	SAMN11943004	PRJNA544527	GCA_008423375.1	◑	2.94	54.5	VVJA01	141	2437	16-Sep-19
112	BIOML-A3	SAMN11943003	PRJNA544527	GCA_008423385.1	◑	2.76	55.1	VVIZ01	26	2224	16-Sep-19
113	BIOML-A5	SAMN11943005	PRJNA544527	GCA_008423405.1	◑	2.76	55.2	VVJB01	24	2222	16-Sep-19
114	BIOML-A2	SAMN11943002	PRJNA544527	GCA_008423445.1	◑	2.76	55.2	VVIY01	23	2231	16-Sep-19
115	BIOML-A1	SAMN11943001	PRJNA544527	GCA_008423455.1	◑	2.76	55.2	VVIX01	31	2230	16-Sep-19
116	S05C.meta.bin_1	SAMN10316581	PRJNA492716	GCA_008671835.1	◔	2.58	56.1	RPXP01	132		23-Sep-19
117	S05B.meta.bin_1	SAMN10316569	PRJNA492716	GCA_008672035.1	◔	2.59	56.1	RPXD01	110		23-Sep-19
118	S05A.meta.bin_2	SAMN10316561	PRJNA492716	GCA_008672205.1	◔	2.6	56.1	RPWV01	104		23-Sep-19
119	D10-15	SAMN10240011	PRJNA486904	GCA_009663785.1	◔	2.74	55.6	RDBQ01	105	2227	14-Nov-19
120	JCM 30893	SAMD00192834	PRJDB8988	GCA_009731575.1	●	2.88	55.59		2	2357	28-Nov-19
121	SC1185_S38_bin.9	SAMN12406434	PRJNA544874	GCA_009773845.1	◑	2.63	55.8	VSMQ01	44		20-Dec-19
122	SA1416_S27_bin.1	SAMN12406435	PRJNA544874	GCA_009773855.1	◑	2.05	56.3	VSMP01	461		20-Dec-19
123	JCM 30893	SAMN13925612	PRJNA603250	GCA_010509235.1	●	2.88	55.6		1	2351	13-Feb-20
124	AK32	SAMN15393864	PRJNA642315	GCA_014170075.1	●	3	55.3		1	2493	12-Aug-20
125	UBG017	SAMN09684600	PRJNA482729	GCA_014848715.1	◔	2.9	55.4	RKGP01	29	2452	5-Oct-20
126	UBG009	SAMN09684595	PRJNA482729	GCA_014848885.1	◔	3.05	58.2	RKGX01	49	2534	5-Oct-20
127	COPD435	SAMN12905382	PRJNA562766	GCA_014871935.1	◔	2.49	55.8	WHIS01	370		8-Oct-20

128	COPD434	SAMN12905381	PRJNA562766	GCA_014871965.1	◔	2.81	55.4	WHIR01	45		8-Oct-20
129	COPD432	SAMN12905379	PRJNA562766	GCA_014871995.1	◔	2.72	55.5	WHIP01	42		8-Oct-20
130	COPD433	SAMN12905380	PRJNA562766	GCA_014872005.1	◔	2.63	55.7	WHIQ01	58		8-Oct-20
131	COPD431	SAMN12905378	PRJNA562766	GCA_014872035.1	◔	2.81	55.4	WHIO01	59		8-Oct-20
132	COPD430	SAMN12905377	PRJNA562766	GCA_014872065.1	◔	2.84	55.3	WHIN01	25		8-Oct-20
133	COPD428	SAMN12905375	PRJNA562766	GCA_014872075.1	◔	2.69	55.7	WHIL01	36		8-Oct-20
134	COPD429	SAMN12905376	PRJNA562766	GCA_014872105.1	◔	2.72	55.3	WHIM01	56		8-Oct-20
135	COPD427	SAMN12905374	PRJNA562766	GCA_014872115.1	◔	2.04	56	WHIK01	530		8-Oct-20
136	COPD426	SAMN12905373	PRJNA562766	GCA_014872125.1	◔	2.75	55.5	WHIJ01	77		8-Oct-20
137	COPD425	SAMN12905372	PRJNA562766	GCA_014872165.1	◔	3.02	58.2	WHII01	106		8-Oct-20
138	Chimpanzee_1.fasta	SAMEA104102601	PRJEB21068	GCA_900184845.1	◑	2.62	55.7	FXXX01	77	2125	29-Jul-17
139	Horse.fasta	SAMEA104102605	PRJEB21068	GCA_900184855.1	◔	2.77	55.7	FXXU01	90	2254	29-Jul-17
140	Chimpanzee_2_1.fasta	SAMEA104102602	PRJEB21068	GCA_900184865.1	◑	2.93	55.9	FXXY01	185	2430	29-Jul-17
141	MOUSE_M89_JASPER.fasta	SAMEA104102609	PRJEB21068	GCA_900184885.1	◑	2.86	55.9	FXZL01	215	2365	29-Jul-17
142	Siamang.fasta	SAMEA104102612	PRJEB21068	GCA_900184905.1	◔	2.65	55.8	FXXV01	110	2139	29-Jul-17
143	Mouse_2_MG.fasta	SAMEA104102608	PRJEB21068	GCA_900184965.1	◑	2.86	55.9	FXYA01	215	2375	29-Jul-17
144	Chimpanzee_2_2.fasta	SAMEA104102603	PRJEB21068	GCA_900184975.1	◑	2.94	55.2	FXXW01	120	2373	29-Jul-17
145	Mouse_1_89.fasta	SAMEA104102607	PRJEB21068	GCA_900184985.1	◔	2.72	55.6	FXYC01	130	2269	29-Jul-17
146	Reindeer.fasta	SAMEA104102611	PRJEB21068	GCA_900185005.1	◑	2.67	55.8	FXYB01	104	2154	29-Jul-17
147	MG2_JASPER.fasta	SAMEA104102606	PRJEB21068	GCA_900185025.1	◑	2.74	55.6	FXXS01	25	2273	29-Jul-17
148	Elephant.fasta	SAMEA104102604	PRJEB21068	GCA_900185045.1	◑	2.67	55.7	FXXZ01	188	2172	29-Jul-17
149	Pig.fasta	SAMEA104102610	PRJEB21068	GCA_900185065.1	◔	2.81	55.8	FXXT01	70	2247	29-Jul-17
150	MGYG-HGUT-02454	SAMEA5851959	PRJEB33885	GCA_902387255.1	●	2.76	55.2		1	2221	9-Aug-19
151	MGYG-HGUT-02452	SAMEA5851957	PRJEB33885	GCA_902387265.1	◔	2.9	58.3	CABMLK01	7	2315	16-Aug-19
152	MGYG-HGUT-02453	SAMEA5851958	PRJEB33885	GCA_902387295.1	◔	3.1	58.1	CABMLH01	15	2512	16-Aug-19

Table S2

Strain	BioSample	BioProject	Assembly	RefSeq FTP	group	Isolate location	Isolate source	Length	GC Content(%)
JCM 30893	SAMD00192834	PRJDB8988	GCA_009731575.1	GCF_009731575.1_ASM973157v1	A	missing	Homo sapiens	2878459	55.64
Chimpanzee_1.fasta	SAMEA104102601	PRJEB21068	GCA_900184845.1	GCF_900184845.1_Chimpanzee_1.fasta	A	na	na	2609266	55.72
Elephant.fasta	SAMEA104102604	PRJEB21068	GCA_900185045.1	GCF_900185045.1_Elephant.fasta	A	na	na	2656729	55.73
Horse.fasta	SAMEA104102605	PRJEB21068	GCA_900184855.1	GCF_900184855.1_Horse.fasta	A	na	na	2767052	55.71
MG2_JASPER.fasta	SAMEA104102606	PRJEB21068	GCA_900185025.1	GCF_900185025.1_MG2_JASPER.fasta	A	na	na	2743798	55.59
Mouse_1_89.fasta	SAMEA104102607	PRJEB21068	GCA_900184985.1	GCF_900184985.1_Mouse_1_89.fasta	A	na	na	2715564	55.64
Pig.fasta	SAMEA104102610	PRJEB21068	GCA_900185065.1	GCF_900185065.1_Pig.fasta	A	na	na	2803504	55.76
Reindeer.fasta	SAMEA104102611	PRJEB21068	GCA_900185005.1	GCF_900185005.1_Reindeer.fasta	A	na	na	2663474	55.75
Siamang.fasta	SAMEA104102612	PRJEB21068	GCA_900184905.1	GCF_900184905.1_Siamang.fasta	A	na	na	2642823	55.76
Urmite	SAMEA3139045	PRJEB6027	GCA_000723745.2	GCF_000723745.2_PRJEB6027_Urmite	A	na	na	2664738	56.03
ATCC BAA-835	SAMN00138213	PRJNA20089	GCA_000020225.1	GCF_000020225.1_ASM2022v1	A	na	na	2664102	55.76
YL44	SAMN03854119	PRJNA289613	GCA_002201495.1	GCF_002201495.1_ASM220149v1	A	Switzerland: Zurich	Mus musculus	2737357	55.66
YL44	SAMN04621615	PRJNA317592	GCA_001688765.2	GCF_001688765.2_ASM168876v2	A	Switzerland: Bern	Mus musculus	2745278	55.66
An78	SAMN06473757	PRJNA377666	GCA_002161325.1	GCF_002161325.1_ASM216132v1	A	not applicable	Gallus gallus	2736972	55.72
EB-AMDK-3	SAMN07980909	PRJNA417203	GCA_003716935.1	GCF_003716935.1_ASM371693v1	A	South Korea: Seoul	Korean adult feces	2663833	55.76
EB-AMDK-4	SAMN07980958	PRJNA417208	GCA_003716955.1	GCF_003716955.1_ASM371695v1	A	South Korea: Seoul	Korean adult feces	2664010	55.76
BSM09	SAMN08162519	PRJNA331216	GCA_002885475.1	GCF_002885475.1_ASM288547v1	A	China	Mus musculus	2645537	55.66
GP02	SAMN08162520	PRJNA331216	GCA_002885465.1	GCF_002885465.1_ASM288546v1	A	China	gut microbiota	2679142	55.72
GP09	SAMN08162521	PRJNA331216	GCA_002885715.1	GCF_002885715.1_ASM288571v1	A	China	gut microbiota	2677689	55.72
GP13	SAMN08162522	PRJNA331216	GCA_002885455.1	GCF_002885455.1_ASM288545v1	A	China	gut microbiota	2679854	55.72
GP14	SAMN08162523	PRJNA331216	GCA_002885425.1	GCF_002885425.1_ASM288542v1	A	China	gut microbiota	2708328	55.53
GP16	SAMN08162524	PRJNA331216	GCA_002885415.1	GCF_002885415.1_ASM288541v1	A	China	gut microbiota	2672910	55.62
GP19	SAMN08162525	PRJNA331216	GCA_002885395.1	GCF_002885395.1_ASM288539v1	A	China	gut microbiota	2790954	55.48

GP20	SAMN08162526	PRJNA331216	GCA_002885375.1	GCF_002885375.1_ASM288537v1	A	China	gut microbiota	2820827	55.62
GP23	SAMN08162527	PRJNA331216	GCA_002885355.1	GCF_002885355.1_ASM288535v1	A	China	gut microbiota	2859287	55.56
GP26	SAMN08162528	PRJNA331216	GCA_002885335.1	GCF_002885335.1_ASM288533v1	A	China	gut microbiota	2679074	55.72
GP27	SAMN08162529	PRJNA331216	GCA_002885315.1	GCF_002885315.1_ASM288531v1	A	China	gut microbiota	2720256	55.51
GP28	SAMN08162530	PRJNA331216	GCA_002885695.1	GCF_002885695.1_ASM288569v1	A	China	gut microbiota	2720086	55.6
GP35	SAMN08162531	PRJNA331216	GCA_002885295.1	GCF_002885295.1_ASM288529v1	A	China	gut microbiota	2861208	55.57
GP40	SAMN08162532	PRJNA331216	GCA_002885675.1	GCF_002885675.1_ASM288567v1	A	China	gut microbiota	2790720	55.48
EB-AMDK-7	SAMN08328879	PRJNA429075	GCA_004015245.1	GCF_004015245.1_ASM401524v1	A	South Korea: Seoul	Korean adult feces	2799431	55.3
139	SAMN10963682	PRJNA523108	GCA_004319565.1	GCF_004319565.1_ASM431956v1	A	China	Mus musculus	2801917	55.74
DSM 22959	SAMN12556229	PRJNA559704	GCA_008000975.1	GCF_008000975.1_ASM800097v1	A	Netherlands	feces	2664043	55.76
JCM 30893	SAMN13925612	PRJNA603250	GCA_010509235.1	GCF_010509235.1_ASM1050923v1	A	na	human feces	2878261	55.64
D10-15	SAMN10240011	PRJNA486904	GCA_009663785.1	GCF_009663785.1_ASM966378v1	A	China:Beijing	ob/ob mouse	2741186	55.57
Chimpanzee_2_2.fasta	SAMEA104102603	PRJEB21068	GCA_900184975.1	GCF_900184975.1_Chimpanzee_2_2.fasta	B	na	na	2931311	55.23
H2	SAMN03278370	PRJNA270907	GCA_004101765.1	GCF_004101765.1_ASM410176v1	B	Belgium: Ghent	Homo sapiens	2819944	55.32
EB-AMDK-1	SAMN07978491	PRJNA417186	GCA_003716915.1	GCF_003716915.1_ASM371691v1	B	South Korea: Seoul	Korean adult feces	2772237	55.39
EB-AMDK-2	SAMN07980963	PRJNA417215	GCA_003716975.1	GCF_003716975.1_ASM371697v1	B	South Korea: Seoul	Korean adult feces	2764211	55.25
GP03	SAMN08162534	PRJNA331216	GCA_002885255.1	GCF_002885255.1_ASM288525v1	B	China	gut microbiota	2824426	55.3
GP04	SAMN08162535	PRJNA331216	GCA_002885655.1	GCF_002885655.1_ASM288565v1	B	China	gut microbiota	2838669	55.32
GP05	SAMN08162536	PRJNA331216	GCA_002885625.1	GCF_002885625.1_ASM288562v1	B	China	gut microbiota	2787215	55.48
GP06	SAMN08162537	PRJNA331216	GCA_002885615.1	GCF_002885615.1_ASM288561v1	B	China	gut microbiota	2770401	55.32
GP21	SAMN08162538	PRJNA331216	GCA_002885235.1	GCF_002885235.1_ASM288523v1	B	China	gut microbiota	2843384	55.37
GP29	SAMN08162539	PRJNA331216	GCA_002885215.1	GCF_002885215.1_ASM288521v1	B	China	gut microbiota	2753166	55.3
GP36	SAMN08162540	PRJNA331216	GCA_002885195.1	GCF_002885195.1_ASM288519v1	B	China	gut microbiota	2750232	55.44
GP38	SAMN08162541	PRJNA331216	GCA_002885595.1	GCF_002885595.1_ASM288559v1	B	China	gut microbiota	2846238	55.24
GP39	SAMN08162542	PRJNA331216	GCA_002885575.1	GCF_002885575.1_ASM288557v1	B	China	gut microbiota	2883372	55.39
GP41	SAMN08162543	PRJNA331216	GCA_002885165.1	GCF_002885165.1_ASM288516v1	B	China	gut microbiota	2770301	55.37

GP43	SAMN08162544	PRJNA331216	GCA_002885155.1	GCF_002885155.1_ASM288515v1	B	China	gut microbiota	2795610	55.57
EB-AMDK-8	SAMN08329008	PRJNA429077	GCA_004015265.1	GCF_004015265.1_ASM401526v1	B	South Korea: Seoul	Korean adult feces	2824041	55.39
EB-AMDK-10	SAMN08329147	PRJNA429085	GCA_004015005.1	GCF_004015005.1_ASM401500v1	B	South Korea: Seoul	Korean adult feces	2763834	55.25
EB-AMDK-11	SAMN08329199	PRJNA429088	GCA_004015025.1	GCF_004015025.1_ASM401502v1	B	South Korea: Seoul	Korean adult feces	2764311	55.26
EB-AMDK-12	SAMN08334970	PRJNA429277	GCA_004015045.1	GCF_004015045.1_ASM401504v1	B	South Korea: Seoul	Korean adult feces	2764297	55.26
EB-AMDK-13	SAMN08334972	PRJNA429279	GCA_004015285.1	GCF_004015285.1_ASM401528v1	B	South Korea: Seoul	Korean adult feces	2763965	55.25
EB-AMDK-14	SAMN08334973	PRJNA429282	GCA_004015065.1	GCF_004015065.1_ASM401506v1	B	South Korea: Seoul	Korean adult feces	2764188	55.25
EB-AMDK-15	SAMN08334975	PRJNA429284	GCA_004015305.1	GCF_004015305.1_ASM401530v1	B	South Korea: Seoul	Korean adult feces	2770098	55.3
EB-AMDK-16	SAMN08334976	PRJNA429285	GCA_004015205.1	GCF_004015205.1_ASM401520v1	B	South Korea: Seoul	Korean adult feces	2770073	55.3
EB-AMDK-17	SAMN08334977	PRJNA429286	GCA_004015225.1	GCF_004015225.1_ASM401522v1	B	South Korea: Seoul	Korean adult feces	2770146	55.3
EB-AMDK-18	SAMN08334978	PRJNA429288	GCA_004015085.1	GCF_004015085.1_ASM401508v1	B	South Korea: Seoul	Korean adult feces	2770124	55.3
CBA5201	SAMN10186677	PRJNA494944	GCA_004104435.1	GCF_004104435.1_ASM410443v1	B	missing	human feces	2860407	55.32
AK32	SAMN15393864	PRJNA642315	GCA_014170075.1	GCF_014170075.1_ASM1417007v1	B	South Korea: Seoul	feces	3004919	55.28
MGYG-HGUT-02454	SAMEA5851959	PRJEB33885	GCA_902387255.1	GCF_902387255.1_UHGG_MGYG-HGUT-02454	C	China	human gut	2762447	55.19
GP01	SAMN08162533	PRJNA331216	GCA_002885265.1	GCF_002885265.1_ASM288526v1	C	China	gut microbiota	2762447	55.19
EB-AMDK-19	SAMN08334979	PRJNA429289	GCA_004015105.1	GCF_004015105.1_ASM401510v1	C	South Korea: Seoul	Korean adult feces	2724248	55.32
EB-AMDK-20	SAMN08334981	PRJNA429292	GCA_004015325.1	GCF_004015325.1_ASM401532v1	C	South Korea: Seoul	Korean adult feces	2724186	55.32
EB-AMDK-21	SAMN08334982	PRJNA429294	GCA_004015345.1	GCF_004015345.1_ASM401534v1	C	South Korea: Seoul	Korean adult feces	2724154	55.32
EB-AMDK-22	SAMN08334983	PRJNA429295	GCA_004015125.1	GCF_004015125.1_ASM401512v1	C	South Korea: Seoul	Korean adult feces	2724161	55.32
am_0171	SAMN10239579	PRJNA496358	GCA_004168105.1	GCF_004168105.1_ASM416810v1	C	USA: Cambridge	Homo sapiens	2772088	55.06
BIOML-A1	SAMN11943001	PRJNA544527	GCA_008423455.1	GCF_008423455.1_ASM842345v1	C	USA:Boston	fecal material	2756409	55.16
BIOML-A2	SAMN11943002	PRJNA544527	GCA_008423445.1	GCF_008423445.1_ASM842344v1	C	USA:Boston	fecal material	2759345	55.16
BIOML-A3	SAMN11943003	PRJNA544527	GCA_008423385.1	GCF_008423385.1_ASM842338v1	C	USA:Boston	fecal material	2761541	55.15
BIOML-A4	SAMN11943004	PRJNA544527	GCA_008423375.1	GCF_008423375.1_ASM842337v1	C	USA:Boston	fecal material	2935244	54.55
BIOML-A5	SAMN11943005	PRJNA544527	GCA_008423405.1	GCF_008423405.1_ASM842340v1	C	USA:Boston	fecal material	2756657	55.16

BIOML-A6	SAMN11943006	PRJNA544527	GCA_008423365.1	GCF_008423365.1_ASM842336v1	C	USA:Boston	fecal material	2763018	55.16
BIOML-A7	SAMN11943007	PRJNA544527	GCA_008423335.1	GCF_008423335.1_ASM842333v1	C	USA:Boston	fecal material	2753636	55.15
BIOML-A8	SAMN11943008	PRJNA544527	GCA_008423325.1	GCF_008423325.1_ASM842332v1	C	USA:Boston	fecal material	2755967	55.15
BIOML-A9	SAMN11943009	PRJNA544527	GCA_008423295.1	GCF_008423295.1_ASM842329v1	C	USA:Boston	fecal material	2979066	54.78
BIOML-A11	SAMN11943011	PRJNA544527	GCA_008423275.1	GCF_008423275.1_ASM842327v1	C	USA:Boston	fecal material	2782022	55.06
BIOML-A13	SAMN11943013	PRJNA544527	GCA_008423265.1	GCF_008423265.1_ASM842326v1	C	USA:Boston	fecal material	2881414	54.67
BIOML-A14	SAMN11943014	PRJNA544527	GCA_008423245.1	GCF_008423245.1_ASM842324v1	C	USA:Boston	fecal material	2876208	54.28
BIOML-A15	SAMN11943015	PRJNA544527	GCA_008423215.1	GCF_008423215.1_ASM842321v1	C	USA:Boston	fecal material	2783632	55.07
BIOML-A16	SAMN11943016	PRJNA544527	GCA_008423185.1	GCF_008423185.1_ASM842318v1	C	USA:Boston	fecal material	2774410	55.07
BIOML-A17	SAMN11943017	PRJNA544527	GCA_008423165.1	GCF_008423165.1_ASM842316v1	C	USA:Boston	fecal material	3050314	54.48
BIOML-A18	SAMN11943018	PRJNA544527	GCA_008423175.1	GCF_008423175.1_ASM842317v1	C	USA:Boston	fecal material	3158556	53.12
BIOML-A19	SAMN11943019	PRJNA544527	GCA_008423135.1	GCF_008423135.1_ASM842313v1	C	USA:Boston	fecal material	2761382	55.11
BIOML-A20	SAMN11943020	PRJNA544527	GCA_008423115.1	GCF_008423115.1_ASM842311v1	C	USA:Boston	fecal material	2751615	55.15
BIOML-A21	SAMN11943021	PRJNA544527	GCA_008423085.1	GCF_008423085.1_ASM842308v1	C	USA:Boston	fecal material	3111566	54.27
BIOML-A22	SAMN11943022	PRJNA544527	GCA_008423095.1	GCF_008423095.1_ASM842309v1	C	USA:Boston	fecal material	2751854	55.15
BIOML-A23	SAMN11943023	PRJNA544527	GCA_008423065.1	GCF_008423065.1_ASM842306v1	C	USA:Boston	fecal material	2752368	55.16
BIOML-A24	SAMN11943024	PRJNA544527	GCA_008423015.1	GCF_008423015.1_ASM842301v1	C	USA:Boston	fecal material	2752383	55.16
BIOML-A25	SAMN11943025	PRJNA544527	GCA_008423035.1	GCF_008423035.1_ASM842303v1	C	USA:Boston	fecal material	2778410	55.04
BIOML-A26	SAMN11943026	PRJNA544527	GCA_008422985.1	GCF_008422985.1_ASM842298v1	C	USA:Boston	fecal material	2752058	55.16
BIOML-A27	SAMN11943027	PRJNA544527	GCA_008422965.1	GCF_008422965.1_ASM842296v1	C	USA:Boston	fecal material	2751903	55.16
BIOML-A28	SAMN11943028	PRJNA544527	GCA_008422995.1	GCF_008422995.1_ASM842299v1	C	USA:Boston	fecal material	2775895	55.06
BIOML-A29	SAMN11943029	PRJNA544527	GCA_008422925.1	GCF_008422925.1_ASM842292v1	C	USA:Boston	fecal material	2751571	55.16
BIOML-A30	SAMN11943030	PRJNA544527	GCA_008422875.1	GCF_008422875.1_ASM842287v1	C	USA:Boston	fecal material	2752525	55.16
BIOML-A31	SAMN11943031	PRJNA544527	GCA_008422895.1	GCF_008422895.1_ASM842289v1	C	USA:Boston	fecal material	2752621	55.16
BIOML-A32	SAMN11943032	PRJNA544527	GCA_008422865.1	GCF_008422865.1_ASM842286v1	C	USA:Boston	fecal material	2752868	55.16
BIOML-A33	SAMN11943033	PRJNA544527	GCA_008422885.1	GCF_008422885.1_ASM842288v1	C	USA:Boston	fecal material	2905849	55

BIOML-A34	SAMN11943034	PRJNA544527	GCA_008422805.1	GCF_008422805.1_ASM842280v1	C	USA:Boston	fecal material	2752298	55.16
BIOML-A35	SAMN11943035	PRJNA544527	GCA_008422795.1	GCF_008422795.1_ASM842279v1	C	USA:Boston	fecal material	2752587	55.16
BIOML-A36	SAMN11943036	PRJNA544527	GCA_008422765.1	GCF_008422765.1_ASM842276v1	C	USA:Boston	fecal material	2753750	55.15
BIOML-A37	SAMN11943037	PRJNA544527	GCA_008422775.1	GCF_008422775.1_ASM842277v1	C	USA:Boston	fecal material	2753150	55.16
BIOML-A38	SAMN11943038	PRJNA544527	GCA_008422815.1	GCF_008422815.1_ASM842281v1	C	USA:Boston	fecal material	2752334	55.16
BIOML-A39	SAMN11943039	PRJNA544527	GCA_008422685.1	GCF_008422685.1_ASM842268v1	C	USA:Boston	fecal material	2755956	55.14
BIOML-A40	SAMN11943040	PRJNA544527	GCA_008422705.1	GCF_008422705.1_ASM842270v1	C	USA:Boston	fecal material	2789753	55
BIOML-A41	SAMN11943041	PRJNA544527	GCA_008422695.1	GCF_008422695.1_ASM842269v1	C	USA:Boston	fecal material	2752380	55.16
BIOML-A42	SAMN11943042	PRJNA544527	GCA_008422715.1	GCF_008422715.1_ASM842271v1	C	USA:Boston	fecal material	2752203	55.16
BIOML-A43	SAMN11943043	PRJNA544527	GCA_008422665.1	GCF_008422665.1_ASM842266v1	C	USA:Boston	fecal material	2752176	55.16
BIOML-A44	SAMN11943044	PRJNA544527	GCA_008422635.1	GCF_008422635.1_ASM842263v1	C	USA:Boston	fecal material	2751304	55.15
BIOML-A45	SAMN11943045	PRJNA544527	GCA_008422615.1	GCF_008422615.1_ASM842261v1	C	USA:Boston	fecal material	2751592	55.16

Table S3

	Number	Length	Gap	AverageLength	N50	N90	MaxLength	MinLength	GC Content(%)
GCF_009731575.1_ASM973157v1	2	2878459	0	1439230	2845645	2845645	2845645	32814	55.64
GCF_000020225.1_ASM2022v1	1	2664102	0	2664102	2664102	2664102	2664102	2664102	55.76
GCF_008000975.1_ASM800097v1	1	2664043	0	2664043	2664043	2664043	2664043	2664043	55.76
GCF_014170075.1_ASM1417007v1	1	3004919	0	3004919	3004919	3004919	3004919	3004919	55.28
GCF_010509235.1_ASM1050923v1	1	2878261	0	2878261	2878261	2878261	2878261	2878261	55.64
GCF_004104435.1_ASM410443v1	1	2860407	0	2860407	2860407	2860407	2860407	2860407	55.32
GCF_004015265.1_ASM401526v1	1	2824041	0	2824041	2824041	2824041	2824041	2824041	55.39
GCF_004319565.1_ASM431956v1	1	2801917	0	2801917	2801917	2801917	2801917	2801917	55.74
GCF_004015245.1_ASM401524v1	1	2799431	0	2799431	2799431	2799431	2799431	2799431	55.3
GCF_003716915.1_ASM371691v1	1	2772237	0	2772237	2772237	2772237	2772237	2772237	55.39
GCF_004015225.1_ASM401522v1	1	2770146	0	2770146	2770146	2770146	2770146	2770146	55.3
GCF_004015085.1_ASM401508v1	1	2770124	0	2770124	2770124	2770124	2770124	2770124	55.3
GCF_004015305.1_ASM401530v1	1	2770098	0	2770098	2770098	2770098	2770098	2770098	55.3
GCF_004015205.1_ASM401520v1	1	2770073	0	2770073	2770073	2770073	2770073	2770073	55.3
GCF_004015025.1_ASM401502v1	1	2764311	0	2764311	2764311	2764311	2764311	2764311	55.26
GCF_004015045.1_ASM401504v1	1	2764297	0	2764297	2764297	2764297	2764297	2764297	55.26
GCF_003716975.1_ASM371697v1	1	2764211	0	2764211	2764211	2764211	2764211	2764211	55.25
GCF_004015065.1_ASM401506v1	1	2764188	0	2764188	2764188	2764188	2764188	2764188	55.25
GCF_004015285.1_ASM401528v1	1	2763965	0	2763965	2763965	2763965	2763965	2763965	55.25
GCF_004015005.1_ASM401500v1	1	2763834	0	2763834	2763834	2763834	2763834	2763834	55.25
GCF_902387255.1_UHGG_MGYG-HGUT-02454	1	2762447	0	2762447	2762447	2762447	2762447	2762447	55.19

GCF_001688765.2_ASM168876v2	1	2745278	0	2745278	2745278	2745278	2745278	2745278	55.66
GCF_004015105.1_ASM401510v1	1	2724248	0	2724248	2724248	2724248	2724248	2724248	55.32
GCF_004015325.1_ASM401532v1	1	2724186	0	2724186	2724186	2724186	2724186	2724186	55.32
GCF_004015125.1_ASM401512v1	1	2724161	0	2724161	2724161	2724161	2724161	2724161	55.32
GCF_004015345.1_ASM401534v1	1	2724154	0	2724154	2724154	2724154	2724154	2724154	55.32
GCF_003716955.1_ASM371695v1	1	2664010	0	2664010	2664010	2664010	2664010	2664010	55.76
GCF_003716935.1_ASM371693v1	1	2663833	0	2663833	2663833	2663833	2663833	2663833	55.76
GCF_002201495.1_ASM220149v1	1	2737357	200	2737357	2737357	2737357	2737357	2737357	55.66
GCF_004101765.1_ASM410176v1	1	2819944	1762	2819944	2819944	2819944	2819944	2819944	55.32
GCF_002885265.1_ASM288526v1	1	2762447	0	2762447	2762447	2762447	2762447	2762447	55.19
GCF_002885165.1_ASM288516v1	14	2770301	6	197878.6	1550586	191472	1550586	256	55.37
GCF_002885295.1_ASM288529v1	14	2861208	35	204372	1495453	130637	1495453	216	55.57
GCF_002884975.1_ASM288497v1	7	2902150	13	414592.9	1484227	491109	1484227	310	58.32
GCF_902387265.1_UHGG_MGYG-HGUT-02452	7	2902150	13	414592.9	1484227	491109	1484227	310	58.32
GCF_002885195.1_ASM288519v1	11	2750232	16	250021.1	1446788	131491	1446788	266	55.44
GCF_002885355.1_ASM288535v1	10	2859287	10	285928.7	824253	177133	1206975	209	55.56
GCF_002885695.1_ASM288569v1	13	2720086	11	209237.4	757281	177296	775863	203	55.6
GCF_002885625.1_ASM288562v1	11	2787215	29	253383.2	703402	143287	825297	394	55.48
GCF_002885515.1_ASM288551v1	14	3197742	82	228410.1	1794655	183394	1794655	1690	57.8
GCF_002885595.1_ASM288559v1	18	2846238	13	158124.3	649895	94596	998758	332	55.24
GCF_002885655.1_ASM288565v1	22	2838669	20	129030.4	630437	64068	856931	257	55.32
GCF_002885095.1_ASM288509v1	15	3096868	6	206457.9	580264	269106	1118826	1426	58.07
GCF_902387295.1_UHGG_MGYG-HGUT-02453	15	3096868	6	206457.9	580264	269106	1118826	1426	58.07
GCF_002885335.1_ASM288533v1	17	2679074	37	157592.6	549589	304816	825374	200	55.72

GCF_002885455.1_ASM288545v1	16	2679854	15	167490.9	548987	105106	609533	347	55.72
GCF_002885675.1_ASM288567v1	12	2790720	12	232560	533417	453876	1206203	244	55.48
GCF_002885235.1_ASM288523v1	18	2843384	12	157965.8	505927	135847	960663	261	55.37
GCF_002884915.1_ASM288491v1	14	3009091	28	214935.1	485960	227833	782796	408	58
GCF_002161325.1_ASM216132v1	10	2736972	0	273697.2	474171	238611	623189	3929	55.72
GCF_002885535.1_ASM288553v1	23	3143021	108	136653.1	455556	154698	972926	237	57.76
GCF_002885575.1_ASM288557v1	23	2883372	37	125364	453623	73360	1094930	209	55.39
GCF_002885395.1_ASM288539v1	10	2790954	8	279095.4	450538	227774	1127989	210	55.48
GCF_002885425.1_ASM288542v1	17	2708328	33	159313.4	534168	118949	843580	255	55.53
GCF_002885465.1_ASM288546v1	20	2679142	71	133957.1	405711	107020	548781	287	55.72
GCF_008423365.1_ASM842336v1	18	2763018	2039	153501	457328	91505	732198	2264	55.16
GCF_008422615.1_ASM842261v1	17	2751592	89	161858.4	383866	88806	729054	3902	55.16
GCF_002885715.1_ASM288571v1	19	2677689	13	140931	377473	133075	719401	669	55.72
GCF_002884995.1_ASM288499v1	21	3188864	57	151850.7	357257	90003	628181	228	57.82
GCF_002885135.1_ASM288513v1	16	2660161	617	166260.1	408879	172976	584044	204	58.12
GCF_002885555.1_ASM288555v1	24	3202443	120	133435.1	355733	109394	685218	243	57.72
GCF_002885375.1_ASM288537v1	15	2820827	43	188055.1	354229	231220	775700	286	55.62
GCF_002885315.1_ASM288531v1	11	2720256	183	247296	518281	312311	926407	1656	55.51
GCF_002885015.1_ASM288501v1	20	3113875	77	155693.8	361569	127174	837066	254	57.81
GCF_008423085.1_ASM842308v1	107	3111566	609	29080.06	325166	8596	504459	1048	54.27
GCF_008423405.1_ASM842340v1	24	2756657	1112	114860.7	384143	87381	730202	1337	55.16
GCF_008422865.1_ASM842286v1	21	2752868	251	131089	383866	88694	504022	1283	55.16
GCF_002885475.1_ASM288547v1	18	2645537	299	146974.3	324554	127106	1086351	979	55.66
GCF_004168105.1_ASM416810v1	41	2772088	1	67611.9	324533	87381	404077	1017	55.06
GCF_008422635.1_ASM842263v1	22	2751304	118	125059.3	324533	89422	503895	1283	55.15
GCF_008422665.1_ASM842266v1	20	2752176	61	137608.8	324533	89422	403642	1283	55.16

GCF_008422685.1_ASM842268v1	24	2755956	187	114831.5	324533	88582	504063	1032	55.14
GCF_008422695.1_ASM842269v1	21	2752380	205	131065.7	324533	88694	504063	1283	55.16
GCF_008422705.1_ASM842270v1	35	2789753	93	79707.23	324533	89422	503871	1089	55
GCF_008422765.1_ASM842276v1	26	2753750	195	105913.5	324533	88467	504115	1032	55.15
GCF_008422775.1_ASM842277v1	21	2753150	285	131102.4	324533	88467	504191	1032	55.16
GCF_008422815.1_ASM842281v1	21	2752334	39	131063.5	324533	88582	504012	1283	55.16
GCF_008422885.1_ASM842288v1	105	2905849	248	27674.75	324533	87381	504050	1002	55
GCF_008422965.1_ASM842296v1	22	2751903	0	125086.5	324533	88513	409301	1032	55.16
GCF_008422985.1_ASM842298v1	22	2752058	324	125093.6	324533	89897	504203	1032	55.16
GCF_008423035.1_ASM842303v1	31	2778410	187	89626.13	324533	89422	504130	1084	55.04
GCF_008423065.1_ASM842306v1	22	2752368	129	125107.6	324533	88467	440089	1283	55.16
GCF_008423175.1_ASM842317v1	49	3158556	234	64460.33	324533	33845	501903	1032	53.12
GCF_008423185.1_ASM842318v1	30	2774410	247	92480.33	324533	88834	503900	1017	55.07
GCF_008423275.1_ASM842327v1	32	2782022	332	86938.19	324533	88467	504224	1032	55.06
GCF_008423325.1_ASM842332v1	26	2755967	149	105998.7	324533	88467	505100	1013	55.15
GCF_008423375.1_ASM842337v1	141	2935244	2244	20817.33	324533	88467	505065	1002	54.55
GCF_008423215.1_ASM842321v1	46	2783632	91	60513.74	324532	88467	500730	1006	55.07
GCF_008423245.1_ASM842324v1	82	2876208	204	35075.71	324532	88467	511387	1005	54.28
GCF_008423295.1_ASM842329v1	121	2979066	252	24620.38	324532	72382	505328	1006	54.78
GCF_008422995.1_ASM842299v1	31	2775895	134	89545	321541	88467	731925	1031	55.06
GCF_008423335.1_ASM842333v1	24	2753636	144	114734.8	324533	88806	403642	1005	55.15
GCF_008423015.1_ASM842301v1	23	2752383	296	119668.8	324533	88582	403643	1032	55.16
GCF_008422895.1_ASM842289v1	24	2752621	276	114692.5	321296	88582	493615	1283	55.16
GCF_008422925.1_ASM842292v1	25	2751571	142	110062.8	321296	88467	449884	1032	55.16
GCF_008422805.1_ASM842280v1	24	2752298	97	114679.1	324533	88467	403643	1032	55.16
GCF_008422875.1_ASM842287v1	23	2752525	440	119675	385341	88582	729054	1283	55.16

GCF_008422715.1_ASM842271v1	22	2752203	214	125100.1	324533	88806	403642	1283	55.16
GCF_008422795.1_ASM842279v1	23	2752587	321	119677.7	324533	88467	403643	1032	55.16
GCF_008423385.1_ASM842338v1	26	2761541	2331	106213.1	324795	90341	414316	1283	55.15
GCF_002885415.1_ASM288541v1	13	2672910	23	205608.5	729631	101481	757984	313	55.62
GCF_008423095.1_ASM842309v1	20	2751854	120	137592.7	324533	89422	403724	1283	55.15
GCF_008423115.1_ASM842311v1	25	2751615	223	110064.6	309661	63828	449884	1283	55.15
GCF_008423265.1_ASM842326v1	77	2881414	88	37420.96	324533	88467	403642	1012	54.67
GCF_002885155.1_ASM288515v1	11	2795610	59	254146.4	310513	162161	780207	1493	55.57
GCF_002885215.1_ASM288521v1	20	2753166	246	137658.3	301342	106430	476533	205	55.3
GCF_002885255.1_ASM288525v1	19	2824426	87	148654	297559	123793	955852	225	55.3
GCF_002885025.1_ASM288502v1	21	3167448	14	150830.9	294414	57449	371330	1510	57.92
GCF_002885075.1_ASM288507v1	27	3102828	10	114919.6	262091	57435	1006086	271	58.05
GCF_002885615.1_ASM288561v1	19	2770401	7	145810.6	256742	141244	484759	997	55.32
GCF_008423135.1_ASM842313v1	25	2761382	70	110455.3	255180	63523	403643	1495	55.11
GCF_008423445.1_ASM842344v1	23	2759345	1220	119971.5	255152	88467	452936	1283	55.16
GCF_008423455.1_ASM842345v1	31	2756409	2228	88916.42	255162	63486	403641	1337	55.16
GCF_008423165.1_ASM842316v1	237	3050314	1654	12870.52	304328	3833	499884	1001	54.48
GCF_900184905.1_Siamang.fasta	47	2642823	0	56230.28	216632	87676	441377	202	55.76
GCF_002885105.1_ASM288510v1	23	2696413	562	117235.4	255858	55369	817845	534	57.77
GCF_900184845.1_Chimpanzee_1.fasta	36	2609266	570	72479.61	194643	87708	396941	204	55.72
GCF_002885055.1_ASM288505v1	42	3174465	22	75582.5	185249	68094	613904	232	57.72
GCF_900184855.1_Horse.fasta	43	2767052	0	64350.05	181961	82762	358849	204	55.71
GCF_900184885.1_MOUSE_M89_JASPER.fasta	73	2837147	709	38865.03	181108	69439	436424	200	55.86
GCF_900184965.1_Mouse_2_MG.fasta	73	2837147	709	38865.03	181108	69439	436424	200	55.86
GCF_900185065.1_Pig.fasta	40	2803504	0	70087.6	177495	88065	344289	200	55.76
GCF_900184865.1_Chimpanzee_2_1.fasta	65	2909581	472	44762.78	172002	68470	436451	200	55.88

GCF_900185045.1_Elephant.fasta	77	2656729	144	34502.97	166248	63887	358906	201	55.73
GCF_000723745.2_PRJEB6027_Urmite	1	2664738	107128	2664738	2664738	2664738	2664738	2664738	56.03
GCF_900185025.1_MG2_JASPER.fasta	25	2743798	1190	109751.9	144033	58377	361782	4954	55.59
GCF_900184975.1_Chimpanzee_2_2.fasta	65	2931311	900	45097.09	155485	45556	362649	201	55.23
GCF_900185005.1_Reindeer.fasta	49	2663474	518	54356.61	134551	58849	251281	200	55.75
GCF_900184985.1_Mouse_1_89.fasta	130	2715564	0	20888.95	126076	24693	410174	201	55.64
GCF_009663785.1_ASM966378v1	105	2741186	0	26106.53	79639	26135	209290	265	55.57
